# A TGF-β-responsive enhancer regulates SRC expression and epithelial–mesenchymal transition-associated cell migration

**DOI:** 10.1242/jcs.261001

**Published:** 2023-08-09

**Authors:** Soshi Noshita, Yuki Kubo, Kentaro Kajiwara, Daisuke Okuzaki, Shigeyuki Nada, Masato Okada

**Affiliations:** ^1^ Department of Oncogene Research, Research Institute for Microbial Diseases, Osaka University, 3-1 Yamadaoka, Suita, Osaka 565-0871, Japan; ^2^ Genome Information Research Center, Research Institute for Microbial Diseases, Osaka University, 3-1 Yamadaoka, Suita, Osaka 565-0871, Japan; ^3^ Human Immunology lab, World Premier International Immunology Frontier Research Centre, Osaka University, Suita, Osaka 565-0871, Japan; ^4^ Laboratory of Oncogene research, World Premier International Immunology Frontier Research Centre, Osaka University, Suita, Osaka 565-0871, Japan; ^5^ Center for Infectious Disease Education and Research, Osaka University, Suita, Osaka 565-0871, Japan

**Keywords:** SRC, TGF-β, Enhancer, Epithelial–mesenchymal transition, Cell migration, Cancer cell

## Abstract

The non-receptor tyrosine kinase SRC is overexpressed and/or hyperactivated in various human cancers, and facilitates cancer progression by promoting invasion and metastasis. However, the mechanisms underlying SRC upregulation are poorly understood. In this study, we demonstrate that transforming growth factor-β (TGF-β) induces SRC expression at the transcriptional level by activating an intragenic the SRC enhancer. In the human breast epithelial cell line MCF10A, TGF-β1 stimulation upregulated one of the *SRC* promotors, the 1A promoter, resulting in increased *SRC* mRNA and protein levels. Chromatin immunoprecipitation (ChIP)-sequencing analysis revealed that the SMAD complex is recruited to three enhancer regions ∼15 kb upstream and downstream of the *SRC* promoter, and one of them is capable of activating the *SRC* promoter in response to TGF-β. JUN, a member of the activator protein (AP)-1 family, localises to the enhancer and regulates TGF-β-induced SRC expression. Furthermore, TGF-β-induced SRC upregulation plays a crucial role in epithelial–mesenchymal transition (EMT)-associated cell migration by activating the SRC–focal adhesion kinase (FAK) circuit. Overall, these results suggest that TGF-β-induced SRC upregulation promotes cancer cell invasion and metastasis in a subset of human malignancies.

## Teaser

**Summary:** TGF-β induces SRC expression at the transcriptional level by activating an intragenic SRC enhancer. TGF-β-induced SRC upregulation then promotes cancer cell invasion and metastasis in a subset of human malignancies.

## INTRODUCTION

The proto-oncogene SRC, a non-receptor tyrosine kinase, plays a pivotal role in regulating various cellular functions including proliferation, survival, adhesion, and migration. SRC facilitates the invasion and metastasis of cancer cells by accelerating their function (Guarino, 2010; Yeatman, 2004). SRC is overexpressed and/or activated in various human malignancies, implying that it plays a crucial role in tumour progression (Ishizawar and Parsons, 2004; Irby and Yeatman, 2000; Verbeek et al., 1996; Wiener et al., 2003; Yeatman, 2004). SRC overexpression was first observed over two decades ago in human breast cancer (Verbeek et al., 1996). Furthermore, a recent multiomics study revealed that SRC is overexpressed at the mRNA level and is essential for breast cancer progression (López-Cortés et al., 2020). However, the mechanisms underlying the overexpression of SRC in breast cancer still remain unclear. In particular, the transcriptional regulation of *SRC* is poorly understood.

Transforming growth factor-β (TGF-β), a multifunctional cytokine, regulates multiple cellular functions, including cell proliferation, differentiation and motility, to maintain tissue homeostasis (Massagué, 2012). Once TGF-β binds to the TGF-β type II receptor, the activated TGF-β type II receptor phosphorylates the TGF-β type I receptor to activate downstream signalling, such as the phosphorylation of the SMAD2 and SMAD3 (SMAD2/3). Phosphorylated SMAD2/3 forms a heterotrimeric complex with SMAD4 (Shi and Massagué, 2003). The SMAD2/3–SMAD4 complex is transported into the nucleus and regulates the expression of target genes by directly binding to regulatory gene sequences in cooperation with other transcription factors and/or coactivators/repressors (the SMAD pathway). TGF-β also activates other signalling pathways, including the extracellular signal-regulated kinase (ERK), c-Jun N-terminal kinase (JNK) and phosphoinositide 3-kinase (PI3K) pathways (non-SMAD pathways) (Colak and ten Dijke, 2017; Gaarenstroom and Hill, 2014).

TGF-β plays a dual role as a tumour suppressor and promoter in cancer. As a tumour suppressor, TGF-β induces cell cycle arrest and apoptosis in normal and premalignant cells to maintain tissue homeostasis. In cancer cells, however, TGF-β promotes tumour progression, including invasion and metastasis, by inducing epithelial–mesenchymal transition (EMT) (Colak and ten Dijke, 2017; Padua and Massagué, 2009). During EMT, epithelial cells lose cell–cell adhesion and apical-basal polarity and acquire mesenchymal traits, such as cell motility and invasiveness (Fuxe et al., 2020). In addition to TGF-β, SRC promotes cell migration and invasion by regulating the turnover of focal adhesions, cytoskeletal remodelling, formation of invadopodia and secretion of matrix metalloproteinases (MMPs) to degrade the extracellular matrix (ECM). Therefore, further studies on the relationship between TGF-β signalling and SRC should be conducted to gain a deeper understanding of cancer progression.

In previous studies, we found that ARHGEF5, a Rho guanine nucleotide exchange factor, is upregulated and promotes cell migration by activating the Rho-ROCK pathway during TGF-β-induced EMT in the human breast epithelial cell line, MCF10A (Komiya et al., 2016; Kuroiwa et al., 2011). Moreover, SRC facilitates the activation of ARHGEF5, leading to cell invasion and tumour growth (Komiya et al., 2016). In addition to these findings, we found that SRC protein and mRNA levels increased, coinciding with TGF-β-induced EMT. However, the mechanisms by which TGF-β signalling regulates SRC gene expression remained unknown.

In this study, we found that a TGF-β-responsive enhancer located in the *SRC* intragenic region upregulated SRC promoter activity via the TGF-β-SMAD and JNK-JUN pathways. We also demonstrated that increased SRC expression is essential for promoting EMT-associated cell motility by phosphorylating focal adhesion kinase (FAK; also known as PTK2). This is the first report to demonstrate the transcriptional regulation of *SRC* induced by an TGF-β-responsive enhancer, which helps to elucidate how SRC is overexpressed in various types of human cancer.

## RESULTS

### SRC protein and exon 1A transcripts are increased during TGF-β-induced EMT

**Fig. 1.**
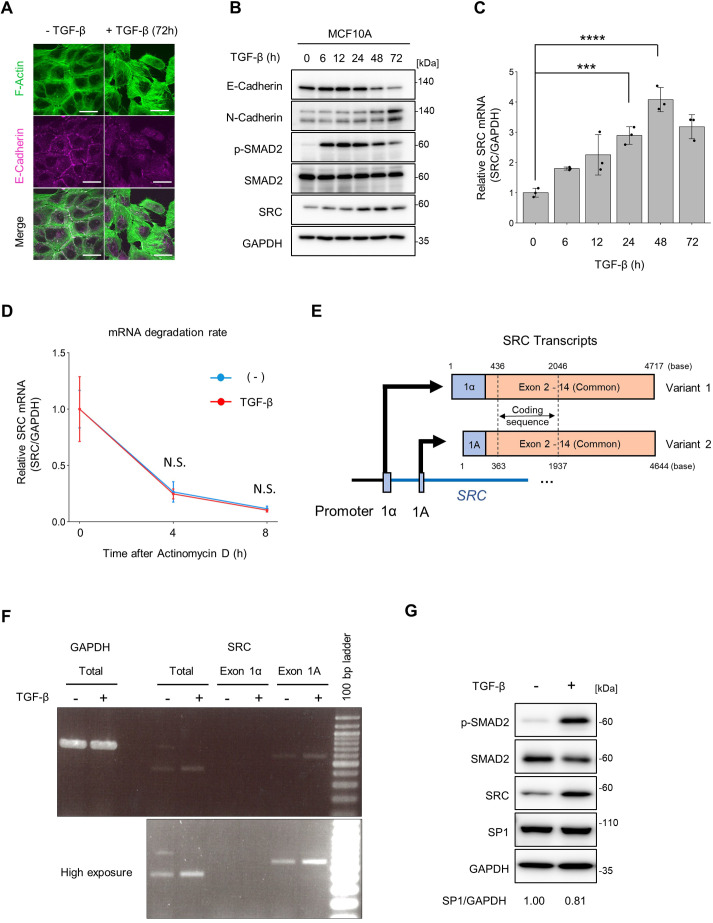
**TGF-β stimulation upregulates *SRC* transcription in MCF10A cells.** (A) MCF10A cells were treated with TGF-β1 (10 ng/ml) for 72 h. The cells were subjected to immunofluorescence staining for F-actin and E-cadherin. Scale bars: 10 µm. (B,C) MCF10A cells were treated with TGF-β1 (10 ng/ml) for the indicated times. (B) Cell lysates were subjected to immunoblotting using the indicated antibodies. (C) Total RNA was isolated and subjected to qPCR. Relative expression levels were calculated using the mean value at 0 h. (D) MCF10A cells were treated with actinomycin D (10 μg/ml) for 4 or 8 h in the presence or absence of TGF-β1 (10 ng/ml). TGF-β stimulation was conducted for 24 h, and then subjected to quantitative real-time PCR. Relative expression levels were calculated using the mean value at 0 h. (E) Schematic diagram of the transcriptional variants of *SRC* mRNA. Each variant is regulated by two different promoters and contains a different exon 1. (F,G) MCF10A cells were treated with TGF-β1 (10 ng/ml) for 24 h. (F) Transcriptional variants of *SRC* mRNA were analysed by reverse transcription-PCR. (G) Immunoblotting analysis for the expression of SP1 in MCF10A cells. The ratio of SP1 to GAPDH is for the blot shown in G, relative to without TGF-β1 (set at 1). All panels show data from at least three experiments; C and D show mean±s.d. ratios obtained from three independent experiments. ***P<0.001; ****P<0.0001; N.S., not significantly different (one-way ANOVA with Tukey's post hoc test).

The normal human breast epithelial cell line, MCF10A, was used to analyse SRC expression during EMT. TGF-β stimulation of MCF10A cells induced morphological changes, such as the reduction of E-cadherin-mediated cell–cell adhesion and the formation of stress fibres (Fig. 1A). In addition, a switch from the cells expressing E-cadherin to expressing N-cadherin was observed, indicating that EMT had been induced in these cells (Fig. 1B). During EMT, SRC protein and mRNA levels were elevated 2- to 4-fold in a time-dependent manner (Fig. 1B,C). In addition to MCF10A cells, TGF-β-induced SRC expression was also observed in the triple-negative breast cancer cell line BT-549 (Fig. S1A,B). SRC has been reported to be overexpressed in renal cancer cell lines owing to downregulation of miR-205, which targets and degrades SRC mRNA (Majid et al., 2011). To verify whether TGF-β affects the stability of SRC mRNA, we determined the degradation rate of SRC mRNA in the presence or absence of TGF-β by arresting transcription using actinomycin D. The result showed that TGF-β treatment did not significantly affect the stability of *SRC* mRNA (Fig. 1D), suggesting that TGF-β regulates *SRC* expression at the transcriptional level.

There are two types of *SRC* transcripts that encode identical SRC proteins whose expression is regulated by two different promoters: SRC1A and SRC1α (Bonham et al., 2000; Ritchie et al., 2000) (Fig. 1E). Reverse transcription PCR (RT-PCR) analysis showed that SRC exon 1A transcripts, but not SRC exon 1α transcripts, were increased by TGF-β stimulation, implying that the SRC1A promoter was selectively activated (Fig. 1F). Previous studies have revealed that SRC1A promoter activity is dependent on the SP1 transcription factor, and an increase in SP1 results in SRC1A promoter activation and SRC expression (Liu et al., 2018; Ritchie et al., 2000). However, TGF-β stimulation did not induce SP1 expression in MCF10A cells (Fig. 1G). Taken together, these results suggest that there are unknown regulatory mechanisms underlying TGF-β-induced SRC transcription.

### SRC expression is directly regulated via the canonical TGF-β-SMAD signalling pathway

**Fig. 2.**
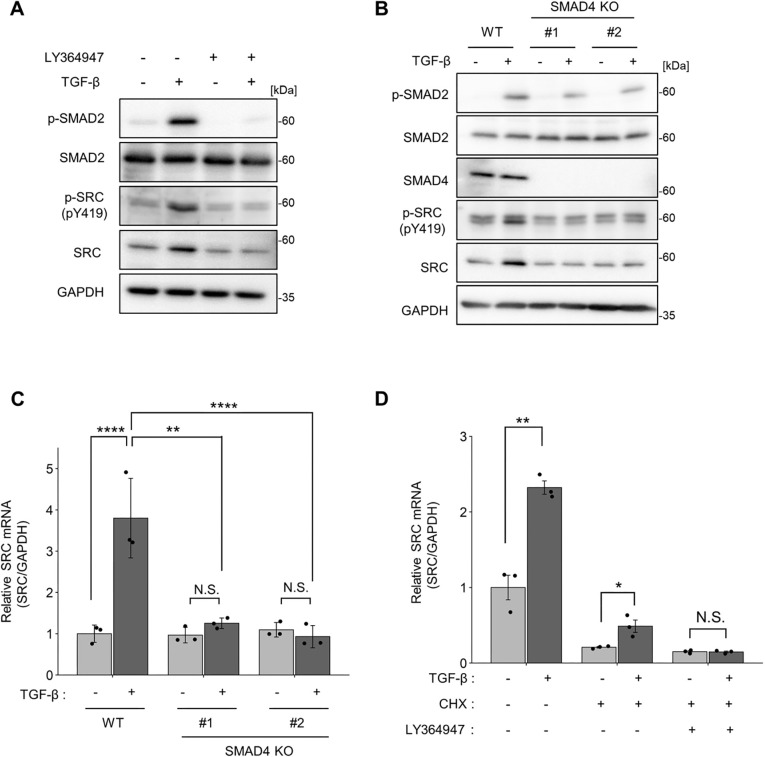
**SRC expression is directly regulated by the TGF-β-SMAD pathway.** (A) MCF10A cells were treated with or without TGF-β1 (10 ng/ml) and the TGFβRI inhibitor LY364947 (2 μM) for 24 h. Cell lysates were subjected to immunoblotting using the indicated antibodies. (B,C) Wild-type (WT) and SMAD4-knockout clones were treated with TGF-β1 (10 ng/ml) for 24 h. (B) Cell lysates were subjected to immunoblotting using the indicated antibodies. (C) Total RNA was isolated and subjected to qPCR. (D) MCF10A cells were pre-treated with cycloheximide (CHX; 10 μg/ml) for 2 h, and then treated with or without TGF-β1 (10 ng/ml) and TGFβRI inhibitor LY364947 (2 μM) for 8 h. Total RNA was isolated and subjected to quantitative real-time PCR. All panels show data from at least three experiments; C and D shown mean±s.d. ratios obtained from three independent experiments. *P<0.05; **P<0.01; N.S., not significantly different [one-way ANOVA with Tukey's post hoc test in (C); unpaired two-tailed *t*-test (D)].

To identify the signalling pathways and transcription factors that induce *SRC* promoter activation, we examined the TGF-β-SMAD signalling pathway. The type I TGF-β receptor inhibitor LY364947 inhibited C-terminal phosphorylation of SMAD2 and TGF-β-induced SRC protein expression (Fig. 2A). We also established SMAD4-knockout (KO) MCF10A cells using CRISPR/Cas9. TGF-β failed to induce SRC expression at the protein and mRNA levels in SMAD4-KO cells, indicating that the SMAD4 is essential for the regulation of SRC transcription (Fig. 2B,C). As TGF-β drives the EMT process by inducing various EMT-associated transcription factors (Fuxe et al., 2020), we also examined the contribution of TGF-β-induced factors. Although inhibition of *de novo* protein synthesis using cycloheximide reduced basal levels of SRC mRNA, TGF-β-induced elevation of SRC transcription was observed even in the presence of cycloheximide and was suppressed by LY364947 (Fig. 2D). These results suggest that SRC transcription is regulated by the pre-existing TGF-β-SMAD pathway.

### *SRC* intragenic enhancer upregulates *SRC* promoter activity upon TGF-β stimulation

**Fig. 3.**
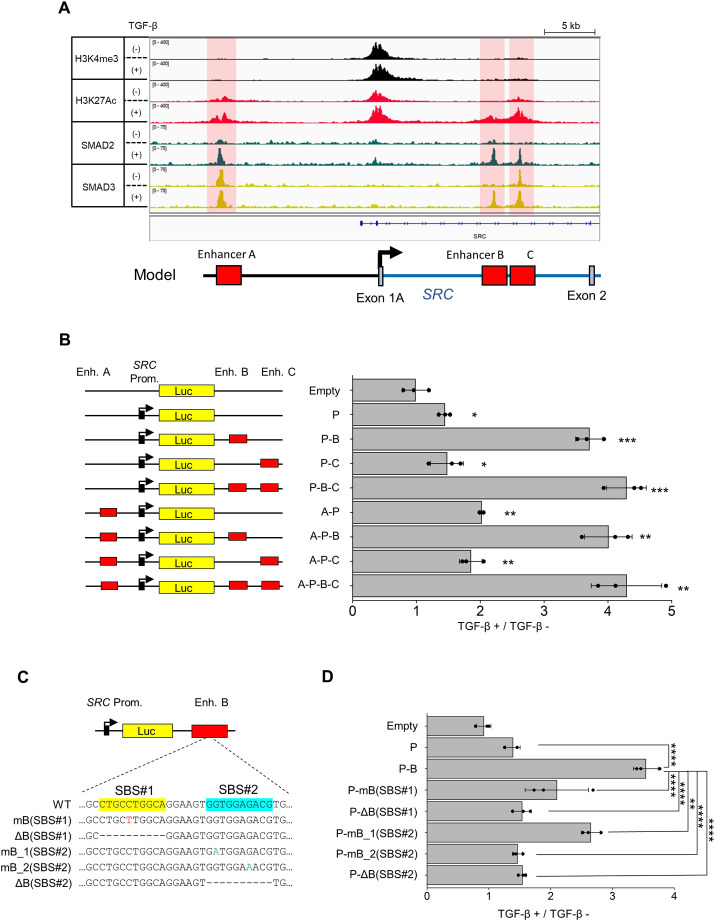
**A TGF-β-responsive SRC intragenic enhancer increases *SRC* promoter activity.** (A) Genomic loci of the *SRC* promoter region. The immunoprecipitation (IP) targets are H3K4me3 (black), H3K27Ac (red), SMAD2 (green) and SMAD3 (yellow). The ChIP-Seq results were visualized using the Integrative Genomics Viewer (IGV) and are representative of one experiment. (B) Schematic diagram of the F-luciferase reporter vectors is shown on the left, and the result of luciferase reporter assay is shown on the right. The reporter vectors and internal control vector were co-transfected in MCF10A cells overnight, and then transfected cells were treated with TGF-β1 (10 ng/ml) for 24 h. Cell lysates were subjected to a luciferase reporter assay. Each data point was normalized to the luminescence of the untreated samples. (C) Schematic diagram of SBS-mutant vector constructs. (D) Luciferase reporter assay conducted using mutant vectors in MCF10A cells. Each data point was normalized to the luminescence of the untreated samples. Data in B and D show mean±s.d. ratios obtained from three independent experiments. *P<0.05; **P<0.01; ***P<0.001; ****P<0.0001 [unpaired two-tailed *t*-test (B); one-way ANOVA with Tukey's post hoc test (D)].

We then attempted to identify the genomic region that contributed to TGF-β-induced SRC transcription using chromatin immunoprecipitation (ChIP) with H3K4me3, H3K27Ac, SMAD2 or SMAD3 antibodies. ChIP-Seq results showed the recruitment of SMAD2 and SMAD3 to three regions ∼15 kb upstream and downstream of the *SRC* promoter, respectively (Fig. 3A; Fig. S1C). Moreover, these regions are marked by H3K27Ac, an epigenetic modification frequently observed at active enhancer sites (Gaarenstroom and Hill, 2014). We refer to these possible regulatory regions as enhancers A, B and C, respectively, from the upstream *SRC* loci. To assess the contribution of these regions to TGF-β-induced *SRC* promoter activation, we measured the activity of these enhancers using a luciferase reporter assay. The results showed that Enhancer B had the ability to upregulate *SRC* promoter activity following TGF-β stimulation (Fig. 3B). Next, we conducted motif analysis using the JASPAR database (Castro-Mondragon et al., 2022) and found that there are two putative SMAD-binding sites (SBS#1 and SBS#2) in enhancer B. To confirm the TGF-β-responsive ability of enhancer B, we introduced mutations into these SBSs by 1 bp substitution or truncation, so that they are not predicted as SMAD-binding sites (Fig. 3C). These mutations reduced TGF-β-induced *SRC* promoter activity, even when another SBS was intact (Fig. 3D; Fig. S1D). These data suggest that the two SBSs in enhancer B are required for the upregulation of *the SRC* promoter activity. Hereafter, we refer to enhancer B as the TGF-β-responsive SRC enhancer (TSE).

### *SRC* promoter activity is also regulated by the TGF-β-JNK-JUN axis

**Fig. 4.**
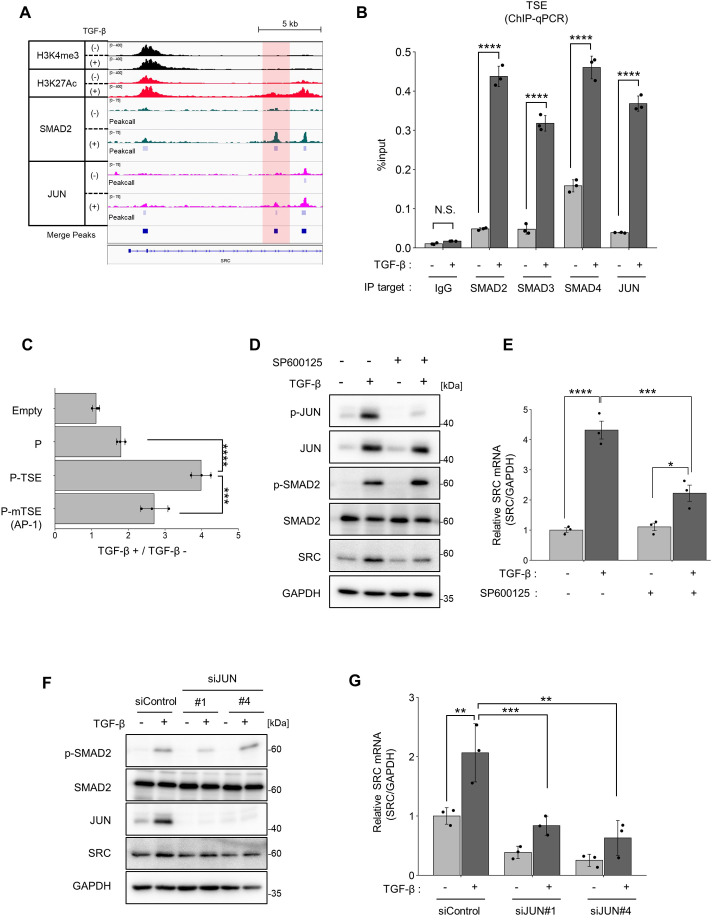
**JUN is required for TSE-mediated *SRC* promoter activation.** (A) Genomic loci of the *SRC* promoter region. The immunoprecipitation (IP) targets are H3K4me3 (black), H3K27Ac (red), SMAD2 (green), and JUN (magenta). The ChIP-Seq results were visualized using the Integrative Genomics Viewer (IGV) and are representative of one experiment. Peak call analysis was conducted using the findPeaks program in HOMER. (B) A ChIP assay was conducted using IgG control or antibodies targeting SMAD2, SMAD3, SMAD4 and JUN. The amount of the TSE region recovered was quantified by qPCR. (C) The result of a luciferase reporter assay with AP-1-binding motif mutant vector. Reporter vectors and internal control vector co-transfected in MCF10A overnight, and transfected cells were treated with TGF-β1 (10 ng/ml) for 24 h. Cell lysates were subjected to a luciferase reporter assay. Each data point was normalized to the luminescence of the untreated samples. (D,E) MCF10A cells were treated with or without TGF-β1 (10 ng/ml) and SP600125 (20 μM) for 24 h. (D) Cell lysates were subjected to immunoblotting using the indicated antibodies. (E) Total RNA was isolated and subjected to qPCR analysis. (F,G) MCF10A cells were treated with the indicated siRNAs overnight and then with or without TGF-β1 (10 ng/ml) for 24 h. (F) Cell lysates were subjected to immunoblotting using the indicated antibodies. (G) Total RNA was isolated and subjected to quantitative real-time PCR. For B, C, E and G, results are mean±s.d. obtained from three independent experiments. **P<0.01; ***P<0.001; ****P<0.0001 [unpaired two-tailed *t*-test in (B); one-way ANOVA with Tukey's post hoc test (C,E,G)].

In addition to SMAD, various transcription factors are required for TGF-β-induced gene expression (Massagué, 2012). Previous studies have shown that cooperation of the AP-1 transcription factor, which consists of JUN and FOS family proteins, plays a pivotal role in breast cancer invasion (Sundqvist et al., 2018, 2020). TGF-β stimulation activates JUN by phosphorylating serine residues via the TGF-β-JNK axis. Moreover, JUN family proteins directly interact with SMAD proteins to regulate downstream gene expression (Liberati et al., 1999), and the AP-1 motif has been shown to be enriched through ChIP-on-chip analysis (Koinuma et al., 2009). Consistent with these studies, motif enrichment analysis using ChIP-Seq for SMAD2 in the presence of TGF-β suggested that AP-1-related motifs were present at the SMAD complex-binding site (Fig. S2A). Furthermore, overlap analysis revealed that over 70% of the SMAD complex-binding regions overlapped with the JUN-binding regions (Fig. S2B). ChIP-Seq and qPCR analyses also revealed the co-localisation of SMAD2, 3 and 4, and JUN in the TSE (Fig. 4A,B). Furthermore, the luciferase reporter assay showed that TGF-β-induced SRC promoter activity was decreased by mutation of the AP-1-binding site in TSE (Fig. 4C).

To further verify the involvement of the JNK-JUN signalling pathway in the regulation of SRC transcription, we examined the effects of JNK inhibitor treatment and JUN protein knockdown. TGF-β stimulation induced the expression and the phosphorylation of JUN protein, and the JNK inhibitor SP600125 inhibited the JNK-mediated phosphorylation of JUN and reduced TGF-β-induced SRC expression (Fig. 4D,E). Moreover, JUN knockdown reduced TGF-β-induced SRC expression (Fig. 4F,G; Fig. S2C). These results demonstrate that *SRC* promoter activity is synergistically regulated by the TGF-β-JNK-JUN axis.

### TSE is essential for TGF-β-induced SRC activation; however, EMT-associated morphological changes occur even in the absence of the TSE

**Fig. 5.**
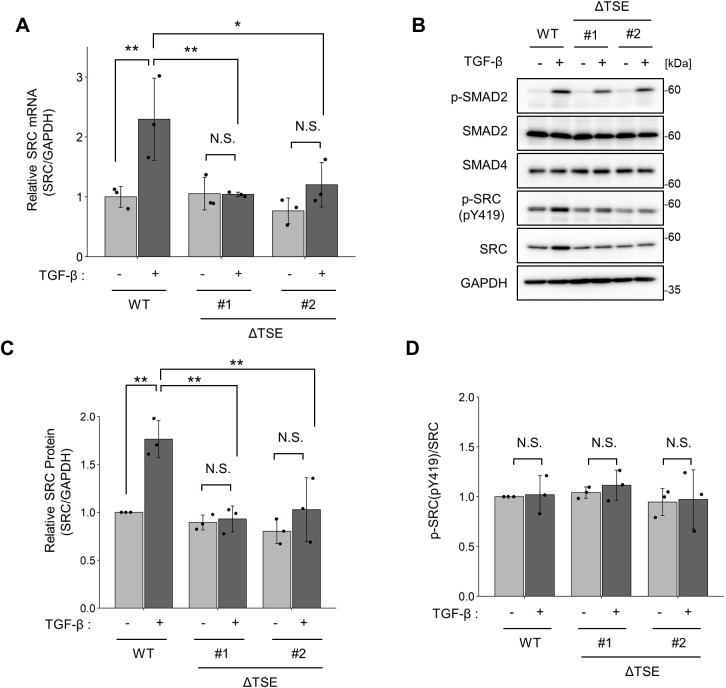
**TGF-β-induced SRC expression and activation are suppressed in TSE-deficient cell lines.** (A,B) Wild-type (WT) MCF10A cells and ΔTSE clones were treated with TGF-β1 (10 ng/ml) for 24 h. (A) Total RNA was isolated and subjected to qPCR. (B) Cell lysates were subjected to immunoblotting using the indicated antibodies. (C,D) Quantification of (C) SRC protein levels and (D) SRC-pY419 in the immunoblot analysis shown in B. For A, C and D, the mean±s.d. ratios were obtained from three independent experiments. *P<0.05; **P<0.01 [N.S., not significantly different; one-way ANOVA with Tukey's post hoc test].

To analyse the effects of TGF-β-induced SRC expression on cellular function, we established TSE-mutant MCF10A cell lines using the CRISPR/Cas9 system (Zientek-Targosz et al., 2008) (Fig. S3A). In these cell lines, TGF-β-induced SRC expression was completely suppressed at both mRNA and protein levels (Fig. 5A–C). These results verify that TSE-mediated SRC upregulation occurs via the endogenous SRC transcriptional machinery. Notably, although the total amount of active SRC (pY419) was increased by TGF-β stimulation in wild-type cells (Fig. 5B), the ratio of active SRC to total SRC did not change in either wild-type or TSE-mutant cells (Fig. 5D). These results suggest that TGF-β-induced SRC activation is mainly attributable to the upregulation of TSE-mediated SRC expression.

**Fig. 6.**
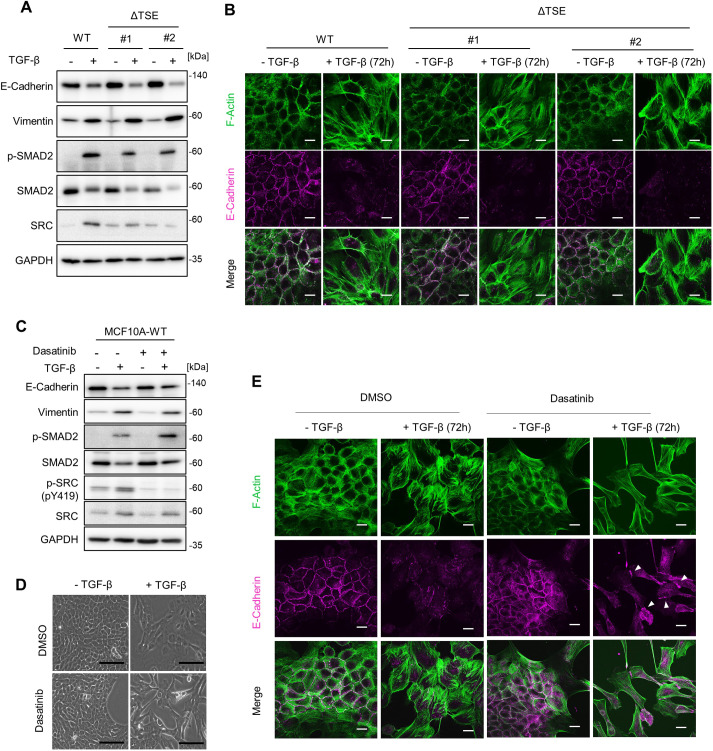
**The effect of TSE mutation or SRC inhibition on TGF-β-induced gene expression and EMT-associated morphological change.** (A,B) Wild-type (WT) MCF10A cells and ΔTSE clones were treated with TGF-β1 (10 ng/ml) for 72 h. (A) Cell lysates were subjected to immunoblotting using the indicated antibodies. (B) Cells were subjected to immunofluorescence staining for F-actin and E-cadherin expression. Scale bars: 10 µm. (C–E) Wild-type MCF10A cells were treated with TGF-β1 (10 ng/ml) in the presence or absence of dasatinib (0.1 μM) for 72 h. (C) Cell lysates were subjected to immunoblotting using the indicated antibodies. (D) Cell morphology observed by phase-contrast microscopy. Scale bars: 20 µm. (E) Cells were subjected to immunofluorescence staining for F-actin and E-cadherin expression. White arrowheads indicate the accumulation of E-cadherin. Scale bars: 10 µm. All panels show data from at least three experiments.

Previous studies have shown that constitutively active SRC (e.g. v-Src or the SrcY527F mutant) promotes the disorganisation of cell–cell contacts by directly phosphorylating E-cadherin and activating the SRC-FAK-ERK-MLCK-myosin pathway, resulting in the induction of EMT (Avizienyte and Frame, 2005; Avizienyte et al., 2004; Fujita et al., 2002; Webb et al., 2004). However, another study has revealed that endogenous SRC activation is not necessary for TGF-β-induced EMT (Maeda et al., 2006). To elucidate the role of SRC in EMT, we evaluated the effects of TGF-β-induced SRC activation on EMT using a TSE-mutant MCF10A cell line. Immunoblot analysis showed that the TSE mutation did not significantly affect the repression of E-cadherin or induction of vimentin expression (Fig. 6A). In addition, EMT-associated cellular traits, including a reduction in E-cadherin-mediated cell–cell adhesion and the formation of stress fibres, were observed in both the wild-type and TSE-mutant cell lines (Fig. 6B). To further characterise these observations, we evaluated the effects of SRC inhibition on EMT marker expression (Fig. 6C) and cell morphology (Fig. 6D,E) induced by TGF-β stimulation. Notably, dasatinib, an SRC inhibitor, did not significantly affect the repression of E-cadherin or induction of vimentin expression, and this immunoblot result is consistent with our findings in the TSE-deficient cell line (Fig. 6A). Regarding E-cadherin expression, a previous study has shown that the SMAD complex and SNAI1 directly bind to the E-cadherin promoter and repress its expression (Vincent et al., 2009). We also found that TGF-β stimulation induced fibroblast-like morphological change in the presence of dasatinib (Fig. 6D). Furthermore, TGF-β-induced reduction of E-cadherin-mediated cell–cell adhesion was also observed even when SRC activity was inhibited (Fig. 6E). Importantly, we also found E-cadherin accumulation at the contact site of the cells (Fig. 6E, white arrowheads in the middle right panel). Previous studies have consistently shown that SRC-mediated endocytosis and degradation are required for the distribution of E-cadherin to the epithelial cell membrane (Balzac et al., 2005; Fujita et al., 2002). Taken together, it is likely that EMT-associated morphological changes and gene expressions are more strongly regulated by TGF-β signalling rather than SRC activity; however, TGF-β-induced SRC activity at the endogenous level is essential for the completion of morphological changes, including the reduction of E-cadherin-mediated cell–cell adhesion in MCF10A cells.

### TSE regulates EMT-associated cell motility by upregulating SRC-mediated FAK phosphorylation

**Fig. 7.**
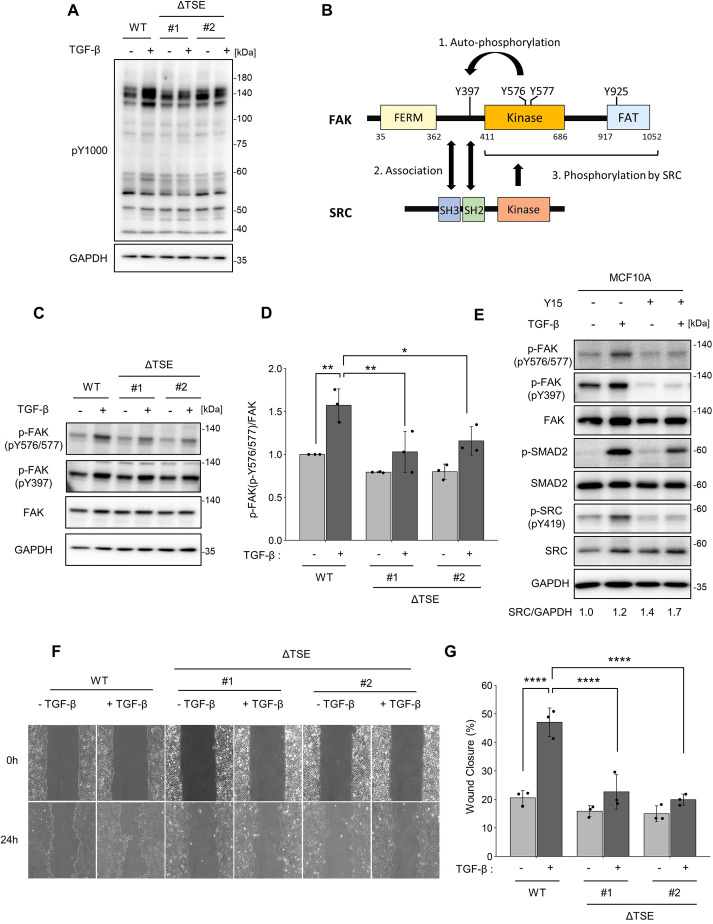
**Deletion of the TSE attenuates SRC-mediated FAK phosphorylation and EMT-associated cell motility.** (A) Wild-type (WT) MCF10A cells and ΔTSE clones were treated with TGF-β1 (10 ng/ml) for 24 h. Cell lysates were subjected to immunoblotting using the indicated antibodies. Image is representative of at least three experiments. (B) Schematic diagram of the interactions between FAK and SRC. (C) Wild-type MCF10A cells and ΔTSE clones were treated with TGF-β1 (10 ng/ml) for 24 h. Cell lysates were subjected to immunoblotting using the indicated antibodies. (D) Quantification of level of FAK phosphorylated at Y576 and/or Y577 (FAK pY576/577) and Y397 (pY397) in immunoblot analysis shown in C. (E) MCF10A cells were treated with or without TGF-β1(10 ng/ml) and the FAK inhibitor Y15 (10 μM) for 24 h. Cell lysates were subjected to immunoblotting using the indicated antibodies. Images is representative from at least three experiments. (F) Wound healing assay of wild-type MCF10A cells and ΔTSE clones treated with or without TGF-β1 (10 ng/ml) for 24 h. (G) Quantification of the wound closure rate using the images shown in F. D and G show mean±s.d. results obtained from three independent experiments. *P<0.05; **P<0.01; ****P<0.0001 (one-way ANOVA with Tukey's post hoc test).

Finally, we investigated the effects of TSE-mediated SRC upregulation on EMT. A cell proliferation assay showed that a growth arrest effect of TGF-β stimulation was observed at the same ratio in both the wild-type and TSE-mutant cell lines, implying that TSE-mediated SRC expression did not significantly affect cell proliferation (Fig. S3B). As the total amount of active SRC increased (Fig. 5B), we attempted to identify proteins phosphorylated by SRC. Immunoblot analysis using an anti-phospho-tyrosine antibody (pY1000) showed that the phosphorylation of ∼130 kDa protein(s) was increased by TGF-β stimulation in the wild-type cell line, but not in TSE mutant cell lines (Fig. 7A). This suggests that TSE-mediated SRC upregulation results in the phosphorylation of these protein(s). We examined several tyrosine-phosphorylated proteins previously reported as SRC substrates (Guarino, 2010) and found that SRC-mediated phosphorylation of FAK was significantly decreased in TSE-mutant cell lines (Fig. 7B–D). Importantly, the FAK inhibitor Y15, which selectively interacts with Y397 in FAK and inhibits autophosphorylation of this residue (Golubovskaya et al., 2008), suppressed SRC-Y419 phosphorylation and subsequent FAK-Y576 and Y577 phosphorylation (Westhoff et al., 2004) (Fig. 7E). This result suggests that TGF-β-induced SRC expression promotes the formation and activation of the SRC-FAK circuit. We also found that the phosphorylation of SMAD2 is partially reduced by Y15 treatment. Previous studies have shown that FAK inhibition or knockdown attenuates TGF-β-induced SMAD phosphorylation, implying crosstalk between TGF-β signalling and FAK activity (Ding et al., 2017; Park et al., 2010). Additionally, a recent study reported that the TGF-β receptor directly interacts with and activates the SRC protein under TGF-β stimulation (Yakymovych et al., 2022). To clarify the detailed mechanisms underlying the activation loop and feedback loop mediated by TGF-β, SRC and FAK, further investigation is needed.

Because SRC-FAK signalling plays a central role in cell migration (Avizienyte and Frame, 2005; Guarino, 2010; Le Coq et al., 2022; Westhoff et al., 2004; Yakymovych et al., 2022), we next examined the effects of TSE-mediated SRC expression and activation on cell motility using a wound healing assay. We performed a wound healing assay using inhibitors of type I TGF-β receptor (LY364947), SRC (dasatinib) and FAK (Y15). TGF-β-induced cell migration was inhibited by the treatment of these inhibitors, suggesting that signalling pathways mediated by type I TGF-β receptor, SRC and FAK are essential for cell migration in MCF10A cells (Fig. S4A,B). Consistent with this, TGF-β-induced cell migration was decreased in the TSE mutant cell lines, (Fig. 7F,G). Furthermore, TGF-β-induced cell motility was recovered upon overexpression of wild-type SRC protein in ΔTSE cell lines, which can bypass TSE-mediated SRC expression (Fig. S4C–E). These results indicate that TSE-mediated SRC upregulation is crucial for EMT-associated cell migration via SRC-FAK signalling.

## DISCUSSION

We investigated the mechanisms underlying TGF-β-induced upregulation of SRC in MCF10A cells and identified a TSE that induces the transcriptional activation of the *SRC* promoter via the TGF-β-SMAD and TGF-β-JNK-JUN signalling pathways. Because SRC has an oncogenic potential, its expression and activity are strictly regulated by post-transcriptional and post-translational pathways. During post-transcriptional regulation, miRNAs directly bind to SRC mRNA, resulting in the degradation and inhibition of SRC mRNA translation (Majid et al., 2011; Okuzaki et al., 2020; Oneyama and Okada, 2015; Wang et al., 2014). Also, the specific activity of SRC is regulated by C-terminal regulatory phosphorylation (Nada et al., 1991; Okada, 2012), and its protein level is regulated by degradation via the lysosome (Tsai et al., 2019) and proteasome (Kim et al., 2004; Mukhopadhyay et al., 2016) systems, or by excretion via small extracellular vesicles (Tanaka et al., 2020). In this study, we focused on the transcriptional regulation of *SRC*. This is the first report demonstrating the transcriptional regulation of *SRC* induced by TGF-β-responsive enhancers.

According to the COSMIC database, SRC is overexpressed in various human cancers; however, mutations in *SRC* are rare (Fig. S5A). Additionally, The Cancer Genome Atlas (TCGA) database analysis revealed that SRC is significantly overexpressed in human breast cancers (Fig. S5B). In colorectal and pancreatic cancers, the TGF-β signalling pathway is frequently abolished by mutations in *SMAD4* or *TGFBR2* (Massagué, 2008; Levy and Hill, 2006). In contrast, TGF-β-SMAD signalling machinery is preserved in breast cancer (Massagué, 2008). Furthermore, TGF-β signalling plays a pivotal role in promoting tumour cell invasion and bone metastasis in breast cancer (Deckers et al., 2006; Sundqvist et al., 2018, 2020). These studies, together with our findings, suggest a possible link between the upregulation of TGF-β signalling and SRC, particularly in breast cancer. Notably, TCGA analysis revealed that SRC was, on average, 1.8-fold overexpressed in patient-derived tissues (Fig. S5B); however, SRC was not overexpressed in various breast cancer cell lines compared to the level seen in the normal breast epithelial cell line MCF10A (Fig. S5C). These results imply that further *in vivo* investigations are needed for a deeper understanding of the mechanisms by which SRC is overexpressed in human cancer tissues, and our findings contribute to the elucidation of this.

A previous study has revealed that TGF-β stimulation rapidly results in 5- to 10-fold activation of SRC in HEK293 T, MEF and PC3 U cells, and that this mechanism does not depend on the activity of the type I TGF-β receptor (Yakymovych et al., 2022). However, in MCF10A cells, SRC was not significantly activated by short-term TGF-β stimulation (Fig. S6A,B). In addition, TGF-β-induced SRC activation depends on TGF-β-SMAD signalling pathway (Fig. 2A,B), and the total amount of active SRC is decreased in TSE-mutant MCF10A cells (Fig. 5B). Moreover, we showed that TGF-β-induced cell migration depended on the activity of type I TGF-β receptor, SRC and FAK in MCF10A cells (Fig. S4A,B). Taken together, it is likely that the TGF-β-SMAD signalling pathway and following TSE-mediated SRC expression, rather than type II TGF-β receptor-dependent SRC activation, are essential for TGF-β-induced cell motility, at least in MCF10A cells. Additionally, we have previously reported that TGF-β2 stimulation induces SRC activation by upregulating the SRC scaffolding protein CASL (also known as NEDD9) without upregulating SRC expression in primary human trabecular meshwork cells (Tsukamoto et al., 2019). Hence, the cell type specificity that determines whether TGF-β can induce SRC expression needs to be further elucidated. In this study, we showed that the AP-1 family protein JUN, in addition to SMAD, binds to the TSE, resulting in *SRC* promoter activation. AP-1, in combination with EGF signalling, plays an essential role in regulating the transcriptional programme of SMAD and promotes TGF-β-induced invasiveness by cooperating with SMAD in the ΔNp63-expressing breast cancer cell lines (Sundqvist et al., 2020). Consistent with that study, we found that co-stimulation with TGF-β and EGF induced SRC expression in normal human epidermal keratinocytes (NHEKs) expressing ΔNp63 (Fig. S7A–C). These results imply that the cooperative mechanisms of SMAD and AP-1 might determine the cell type specificity of TGF-β-induced SRC expression. In addition, TSE contains has the H3K4me1 but not H3K27Ac epigenetic modifications, which represents a poised enhancer in the basal state of MCF10A and NHEK cells (Fig. S7D). This suggests that cell type-specific epigenetic states might also determine cell type specificity of TGF-β-induced SRC expression.

We also found that TSE-mediated SRC expression was crucial for increased FAK phosphorylation and TGF-β-induced cell motility. SRC-FAK signalling accelerates tumour invasiveness by regulating integrin-mediated cell adhesion, cell motility and ECM degradation. Therefore, the TSE-mediated activation of the SRC-FAK circuit might promote EMT-associated cancer cell migration and invasion. As SRC is a key protein in tumour progression, various small-molecule inhibitors of SRC have been developed for anti-cancer therapy (Mayer and Krop, 2010). However, ATP-competitive SRC inhibitors induce conformational changes in SRC, leading to a stable interaction with FAK at focal adhesions. A subsequent reduction in the inhibitor concentration enables SRC to readily phosphorylate FAK and, paradoxically, activate the FAK-Grb2-Erk signalling pathway (Higuchi et al., 2021). In this study, we demonstrated that the inhibition of TGF-β-induced SRC expression results in the suppression of SRC activity and EMT-associated cell migration. This result suggests that the machinery of TSE-mediated SRC transcription could be a novel inhibitory target of SRC that does not cause unexpected effects, such as conformational changes in SRC.

In summary, we demonstrate that a TGF-β-responsive enhancer upregulates SRC expression and that TGF-β-induced SRC expression is essential for EMT-associated cell migration. These findings raise the possibility that the overexpression of SRC observed in a subset of human cancers is induced by TGF-β secreted in the tumour microenvironment. Further investigation into the transcriptional mechanisms of *SRC* might provide promising targets for anti-cancer drugs that prevent tumour invasion and metastasis.

## MATERIALS AND METHODS

### Cell culture

MCF10A cells (ATCC) were cultured in DMEM/F12 (Nacalai Tesque) supplemented with 5% horse serum (Gibco), 20 ng/ml EGF (PeproTech), 0.5 µg/ml hydrocortisone (Sigma), 10 µg/ml insulin (Wako), 100 ng/ml Cholera toxin (Bio Academia), and a 1% penicillin-streptomycin solution (Nacalai Tesque). MCF7, T47D, MDA-MB-231 cells (ATCC) and Hs578T cells (JCRB) were cultured in DMEM supplemented with 10% fetal bovine serum (FBS). BT-549 cells (JCRB) were cultured in RPMI1640 (Nacalai Tesque) containing 10% FBS, 0.023 U/ml insulin (Wako), and a 1% penicillin-streptomycin solution (Nacalai Tesque). Normal human epidermal keratinocyte (NHEK) cells were purchased from PromoCell and cultured in keratinocyte growth medium 2 (PromoCell). The cells were maintained in a CO_2_ incubator at 37°C and 5% CO_2_. Recombinant human TGF-β1 (CHO derived) was purchased from PeproTech.

### Antibodies and inhibitors

The following primary antibodies were used in this study: anti-v-Src (OP07; 1:1000 for WB) antibody was purchased from Sigma-Aldrich. Anti-phospho-Src family (pY416) (2101; 1:500 for WB), anti-phospho-tyrosine (8954; 1:1000 for WB), anti-SMAD2 (5339; 1:1000 for WB and 1:50 for ChIP), anti-phospho-SMAD2 (3108; 1:1000 for WB), anti-SMAD3 (9523; 1:1000 for WB and 1:50 for ChIP), anti-c-Jun (9165; 1:1000 for WB and 1:50 for ChIP), anti-p-c-Jun (3270; 1:1000 for WB), anti-vimentin (5741; 1:1000 for WB), anti-p63-α (13109; 1:1000 for WB), anti-H3K4me3 (9751; 1:50 for ChIP), and anti-H3K27Ac (8173; 1:100 for ChIP) were purchased from Cell Signaling Technology. The anti-E-cadherin (610181; 1:1000 for WB and 1:500 for IF), anti-N-cadherin (610920; 1:1000 for WB), and anti-FAK (610087; 1:1000 for WB) antibodies were purchased from BD Biosciences. Anti-GAPDH (32233; 1:2500 for WB), anti-SP1 (59; 1:1000 for WB), anti-SMAD4 (7966; 1:1000 for WB and 1:50 for ChIP), and anti-phospho-FAK (pY576/577; 1:1000 for WB) (21831) antibodies were purchased from Santa Cruz Biotechnology. Anti phospho-FAK (pY397; 1:1000 for WB) (700255) antibody was purchased from Thermo Fisher Scientific.

The following inhibitors were used in this study: TGFβR-I inhibitor LY364947 (S2805), JNK inhibitor SP600125 (S1460) and FAK inhibitor Y15 (S5321) were purchased from Selleck. Dasatinib (ab142050) was obtained from Abcam. Mitomycin C Solution (20898-21) was purchased from Nacalai Tesque. Actinomycin D (018-21264) was purchased from Wako. Concentration used and time of incubation are given in figure legends.

### Plasmid construction and gene transfer

The DNA sequences of enhancers A, B and C were cloned by PCR using human genomic DNA as the template and subcloned into the pGV-B2 plasmid (Toyo Bnet). TSE mutants were generated using mutagenesis PCR. Wild-type SRC was generated by PCR using human cDNA and were subcloned into a pCX4 retroviral plasmid (generously donated by Dr Tsuyoshi Akagi, Eisai Co., Ltd.). Gene transfer of pCX4 was mediated by retroviral infection. Plasmids were transfected using PEI MAX (Polysciences, Inc.). Primers used in this experiment are listed in Table S1. siRNAs were purchased from Sigma-Aldrich and transfected using Lipofectamine RNAiMAX (Thermo Fisher Scientific). The siRNAs used in this study are listed in Table S2.

### Immunoblotting

Cells were lysed with RIPA buffer (50 mM Tris-HCl pH 7.4, 150 mM NaCl, 1% NP-40, 0.1% SDS, 1 mM EDTA, 0.5% sodium deoxycholate, 1 mM Na_3_VO_4_, 20 mM NaF, 1 mM PMSF and protease inhibitor cocktail (Nacalai Tesque)], and debris were removed by centrifugation at 15,000 ***g*** for 10 min. Horseradish peroxidase-conjugated anti-mouse or anti-rabbit IgG (Zymed Laboratories Inc.) was used as the secondary antibody. All immunoblots were visualised using a Luminograph II System (Atto) and quantified using ImageJ software. Original images for blots presented in this paper are shown in Fig. S8.

### Immunofluorescence microscopy

The cells were grown on type I collagen-coated coverslips, fixed with 4% paraformaldehyde and permeabilised with PBS containing 0.03% Triton X-100. The permeabilised cells were blocked with Blocking One (Nacalai Tesque) and incubated overnight with primary antibodies, followed by incubation with Alexa Fluor 488-conjugated phalloidin and Alexa Fluor 594-conjugated secondary antibodies. Immunostained samples were observed using an FV1000 confocal microscope (Olympus).

### RNA isolation and qRT-PCR

Total RNA was isolated from the cells using a NucleoSpin RNA kit (Macherey-Nagel), and cDNA was prepared using the ReverTra Ace RT Master Mix (TOYOBO) according to the manufacturer's instructions. Real-time PCR was performed using a QuantStudio 5 Real-Time PCR System (Applied Biosystems) and THUNDERBIRD NEXT SYBR qPCR Mix (TOYOBO). Gene expression levels were normalised to GAPDH expression levels. The primers used for this analysis are listed in Table S3.

### CRISPR/Cas9-based gene knockout and mutagenesis

Sequences of gRNA were designed using CRISPRdirect (DBCLS) and inserted into pX458 (Addgene #48138). The Target-gRNA containing pX458 was transfected into MCF10A cells using PEI MAX (Polysciences, Inc.). The following day, EGFP-positive cells were isolated using a FACSAria III sorter (BD Biosciences). Genomic mutations were confirmed by immunoblotting and sequencing. Sequences of the gRNA and primers used for genotyping are listed in Table S4.

### ChIP-Sequencing and data processing

Cells were cultured in a 15 cm dish to 80–90% confluence (∼2×10^7^ cells), and one dish was used for immunoprecipitation. ChIP experiments were performed using the SimpleChIP Enzymatic Chromatin IP kit (9003, Cell Signalling Technologies) according to the manufacturer's instructions. The antibodies used for ChIP were anti-H3K4me3 (9751; 1:50 for ChIP), anti-H3K27Ac (8173; 1:100 for ChIP), anti-Smad2 (5339; 1:50 for ChIP), anti-Smad3 (9523; 1:50 for ChIP), anti-Smad4 (46535; 1:50 for ChIP), anti-c-Jun (9165; 1:50 for ChIP) and anti-JunB (3753; 1:50 for ChIP) purchased from Cell Signaling Technology.

ChIP-seq libraries were prepared using the KAPA Hyper Preparation Kit. Sequencing was performed on a NovaSeq 6000 platform in 101+101 base paired-end mode. Illumina-TruSeq Adapter Trimming was performed on ChIP-seq reads, using cutadapt v2.7, discarding reads left with <30 bp. ChIP-seq reads were aligned to the UCSC hg19 genome using Bowtie2 version 2.3.5.1. BigWig files were generated using the bamCoverage software of the deepTools package. The ChIP-Seq results were visualized using the Integrative Genomics Viewer (IGV) version 2.8.0. (https://software.broadinstitute.org/software/igv/). Data collected is available from the Gene Expression Omnibus (GEO) accession number GSE216432.

Peak call analysis, merge peak analysis and find motif analysis were performed by ‘findPeaks’ program, ‘mergePeaks’ program and ‘findMotifsgenome.pl’ program, respectively, in the HOMER (http://homer.ucsd.edu/homer/index.html) version 4.11 package. ChIP-Seq data for H3K4me1 in MCF10A cells were obtained from the GSE85158 dataset. ChIP-seq data for SMAD2/3 in BT-549 cells were obtained from the GSE104352 dataset. ChIP-seq data for H3K4me1 and H3K27Ac in NHEK were obtained from GSE29611.

### Luciferase reporter assay

For cell preparation, 5×10^4^ cells were seeded in 48-well plates and cultured for 24 h. Cells were co-transfected with the pGV-B2 reporter vector and pRL-TK internal control vector (Toyo Bnet) for 20 h. The following day, cells were stimulated with TGF-β for 24 h by refreshing the medium with or without 10 ng/ml TGF-β, and luciferase activity was assayed using a PicaGene Dual Sea Pansy Luminescence Kit (Toyo Bnet) according to the manufacturer's instructions.

### Cell proliferation assay

For cell preparation, 1×10^3^ cells were seeded in 96-well plates and cultured overnight. The following day, cells were stimulated with 10 ng/ml TGF-β and cultured for 1–4 days. Cell counting was performed using the Cell Count Reagent SF (Nacalai Tesque) before TGF-β stimulation (day 0) and at each time point of 1–4 days after TGF-β stimulation. After adding the reagent to the medium, cells were incubated for 1 h in a CO_2_ incubator, and then the absorbance at 450 nm was measured using a Multiskan FC (Thermo Fisher Scientific).

### Wound healing assay

For cell preparation, 3×10^4^ cells were seeded into a two-well silicone culture insert (ib80209, Ibidi) in a 35 mm dish and cultured overnight. To stop cell proliferation, cells were treated with 10 µg/ml mitomycin C-containing medium for 2 h, and then the culture inserts were removed. Cells were washed twice with PBS and cultured in a medium with or without 10 ng/ml TGF-β for 24 h. Cell images were taken at 0 h, 12 h and 24 h time points.

### Statistics

All statistical analyses were performed using R (https://www.r-project.org/) version 4.0.3 and Microsoft Excel. One-way analysis of variance (ANOVA) with Tukey's post-hoc test was used for multiple group comparisons. Unpaired two-tailed *t*-tests were performed for comparisons between the two groups. A *P*-value less than 0.05 was considered significant. All data were obtained from at least three independent experiments.

## Supplementary Material

10.1242/joces.261001_sup1 Supplementary information
