# Supplementary material for: A TGF-β-responsive enhancer regulates SRC expression and epithelial–mesenchymal transition-associated cell migration
Source: J Cell Sci. 2023 Aug 9;136(15):jcs261001. doi: 10.1242/jcs.261001 (PMC10445741; doi:10.1242/jcs.261001)
Supplement: Supplementary information [file joces-136-261001-s1.pdf]

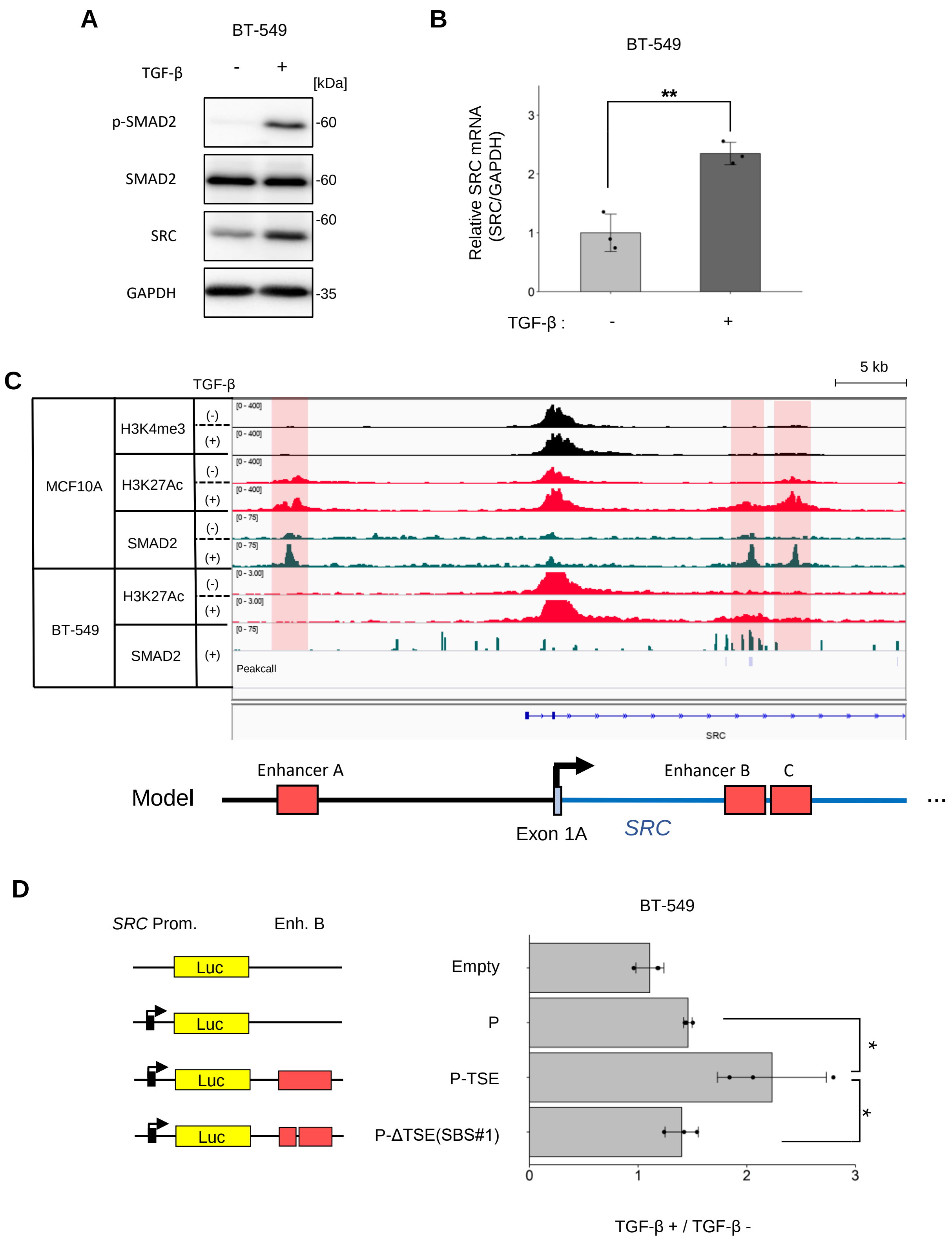

Fig. S1. TGF-β stimulation upregulates *SRC* transcription in triple negative breast cancer cell line BT-549.

(A, B) triple-negative breast cancer cell line BT-549 was treated with TGF-β1 (10 ng/ml) for 24h. (A) Cell lysates were subjected to immunoblotting using the indicated antibodies. (B) Total RNA was isolated and subjected to qPCR. (C) Genomic loci of the *SRC* promoter region. The IP targets are H3K4me3 (black), H3K27Ac (red), and SMAD2 (green). The ChIP-Seq results in MCF10A and BT-549 cells were visualized using IGV. Peak call analysis was conducted using the findPeaks program in HOMER. (D) Luciferase reporter assay was conducted using the SBS mutant vector in BT-549 cells. Each data point was normalized to the luminescence of the untreated samples. (B, D) Mean ratios ± SDs were obtained from three independent experiments. * $p < 0.05$; ** $p < 0.01$; Unpaired two-tailed *t*-test. in (B); One-way ANOVA with Tukey's post hoc test in (D).

Journal of Cell Science • Supplementary information

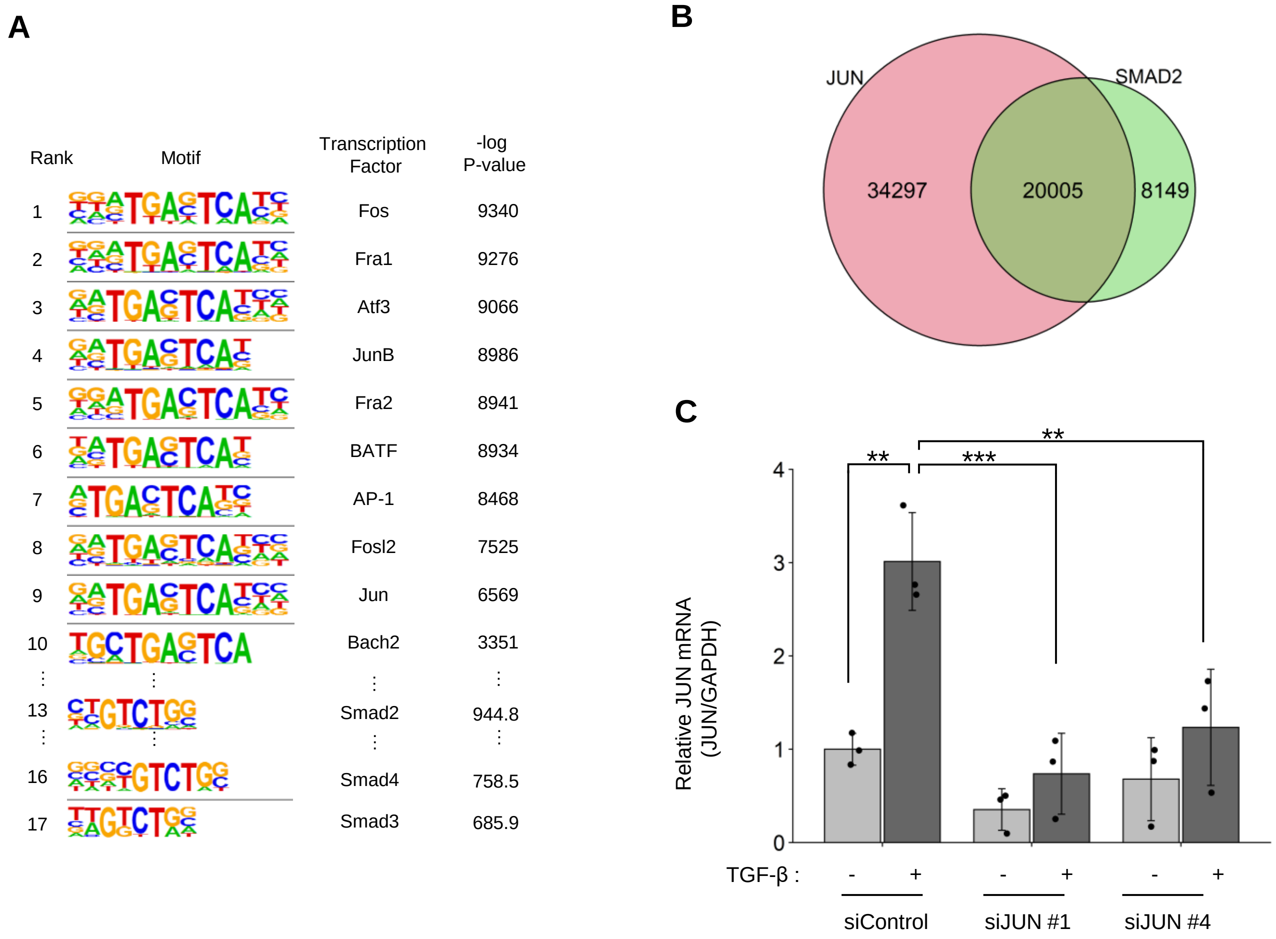

Fig. S2. Motif enrichment analysis focusing on JUN, and knock down of JUN by siRNAs in MCF10A.

(A) Motifs enriched in the SMAD complex binding site in TGF-β-stimulated MCF10A cells. (B) Comparison of the locus of JUN binding sites and SMAD complex binding sites. (C) MCF10A cells were treated with the indicated siRNAs overnight and then with or without TGF-β1(10 ng/ml) for 24h. Total RNA was isolated and subjected to qPCR. (C) Mean ratios ± SDs were obtained from three independent experiments. **, $p < 0.01$; ***, $p < 0.001$; One-way ANOVA with Tukey's post hoc test.

Journal of Cell Science • Supplementary information

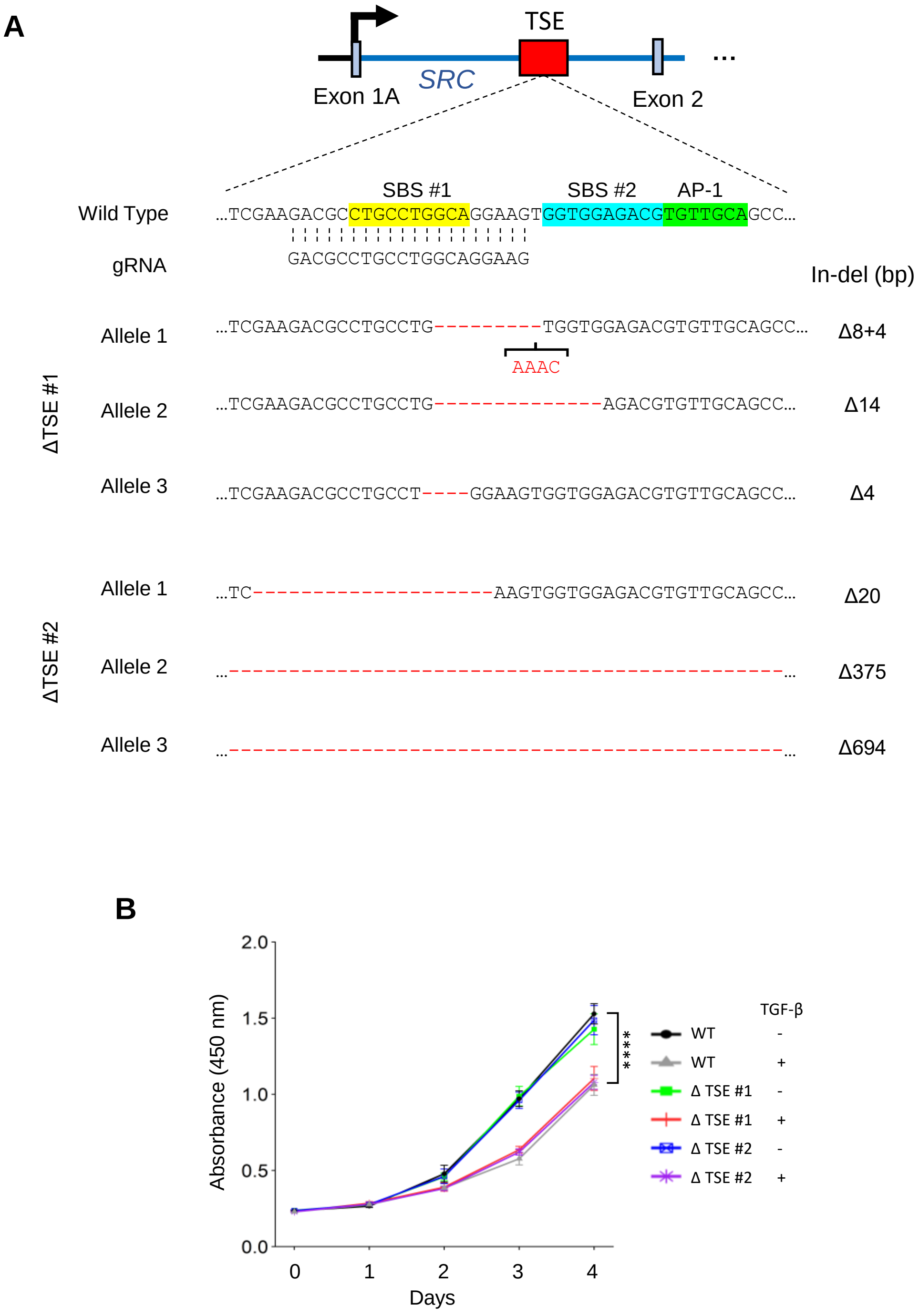

Fig. S3. DNA sequences in ΔTSE MCF10A cell lines and TGF-β reduced cell proliferation rate in both WT and ΔTSE cell lines.
(A) Schematic diagram of CRISPR/Cas9-based mutagenesis of TSE. The DNA sequence in TSE mutant clones are shown in the middle and bottom. (B) Cell proliferation assay conducted using wild-type and ΔTSE MCF10A cells. Wild-type cells and ΔTSE cells were treated with or without TGF-β1 (10 ng/ml) from the day 0 point and cultured for 4 days. Mean ratios ± SDs were obtained from five biologically independent samples. Representative result from three independent experiments are shown. ****, $p < 0.0001$; Unpaired two-tailed *t*-test.

Journal of Cell Science • Supplementary information

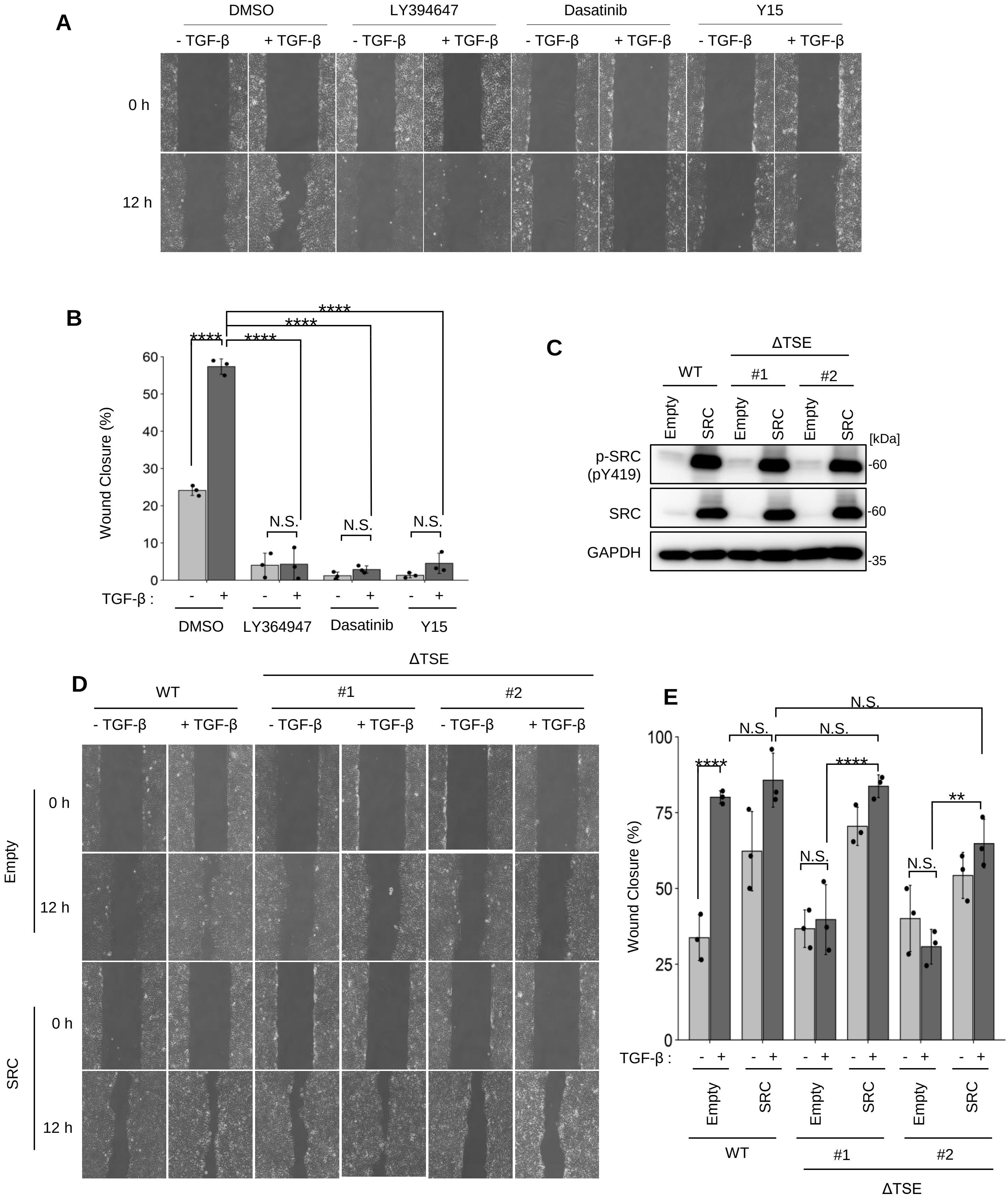

Fig. S4. The effect of the TβRI, SRC, and FAK inhibitors, or overexpression of SRC in MCF10A
(A) Wound healing assay of wild-type MCF10A cells treated with or without TGF-β1 (10 ng/ml) in the presence or absence of DMSO, LY394647, dasatinib, or Y15 for 12h. (B) Quantification of the wound closure rate using the images shown in (A). (C) SRC is overexpressed in wild-type and ΔTSE MCF10A. Cell lysates were subjected to immunoblotting using the indicated antibodies. (D) Wound healing assay using SRC overexpressed MCF10A cells treated with or without TGF-β1 (10 ng/ml) for 12h. (E) Quantification of the wound closure rate using the images shown in (D). (B, E) The mean ratios ± SDs were obtained from three independent experiments. **, $p < 0.01$; ****, $p < 0.0001$; One-way ANOVA with Tukey's post hoc test.

Journal of Cell Science • Supplementary information

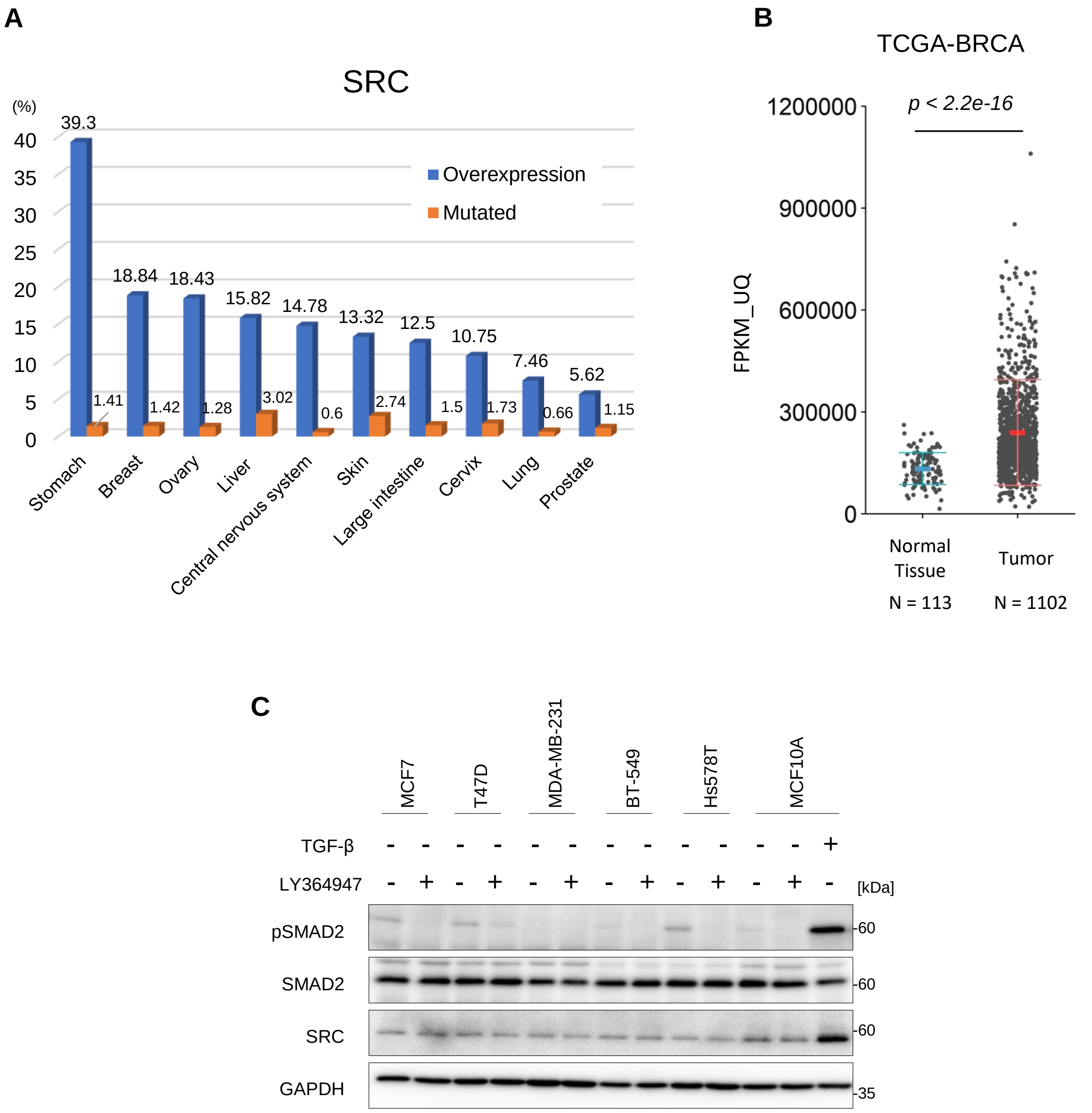

Fig. S5. SRC is overexpressed in various types of human cancers, but not in breast cancer cell lines. (A) The frequencies of somatic mutations in *SRC* in various human cancers were obtained from the Catalogue of Somatic Mutations in Cancer (COSMIC; https://cancer.sanger.ac.uk/cosmic). Blue and orange bars indicate the ratios of overexpression and genetic mutations, respectively. (B) SRC mRNA expression was analyzed using a cohort of breast cancer patients from The Cancer Genome Atlas (TCGA-BRCA). The *p*-value was determined using the Wilcoxon rank-sum test. (C) Cells were treated with TGFβRI inhibitor LY364947 (2 µM) or TGF-β1(10 ng/ml) for 48h. Cell lysates were subjected to immunoblotting using the indicated antibodies.

Journal of Cell Science • Supplementary information

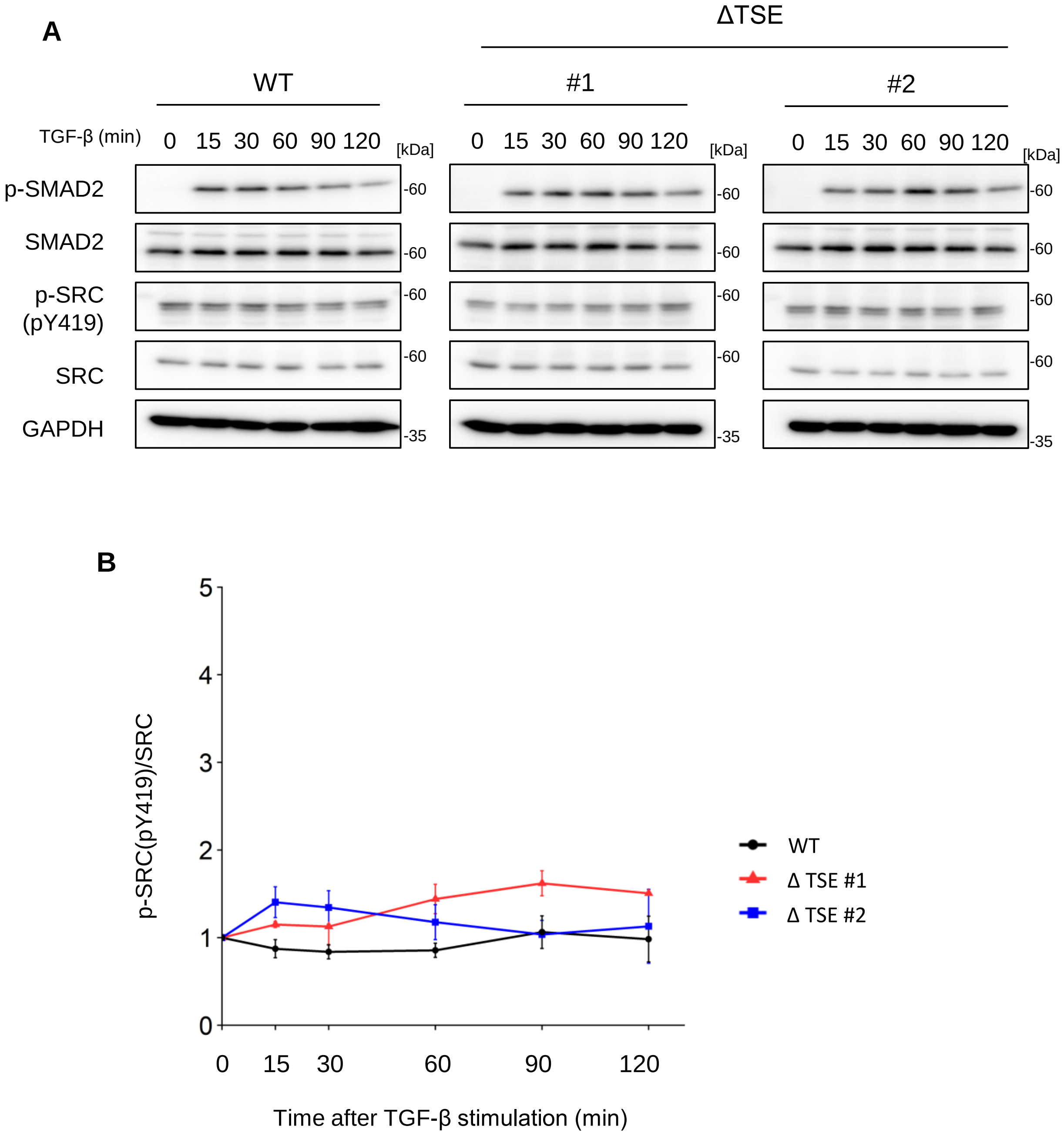

Fig. S6. Short-time TGF-β stimulation in wild-type and ΔTSE cell lines.
(A, B) MCF10A cells were treated with TGF-β1 (5 ng/ml) for the indicated times. (A) Cell lysates were subjected to immunoblotting using the indicated antibodies. (B) Quantification of SRC-pY419 in the immunoblot analysis shown in (A). (B) The mean ratios ± SDs were obtained from three independent experiments.

Journal of Cell Science • Supplementary information

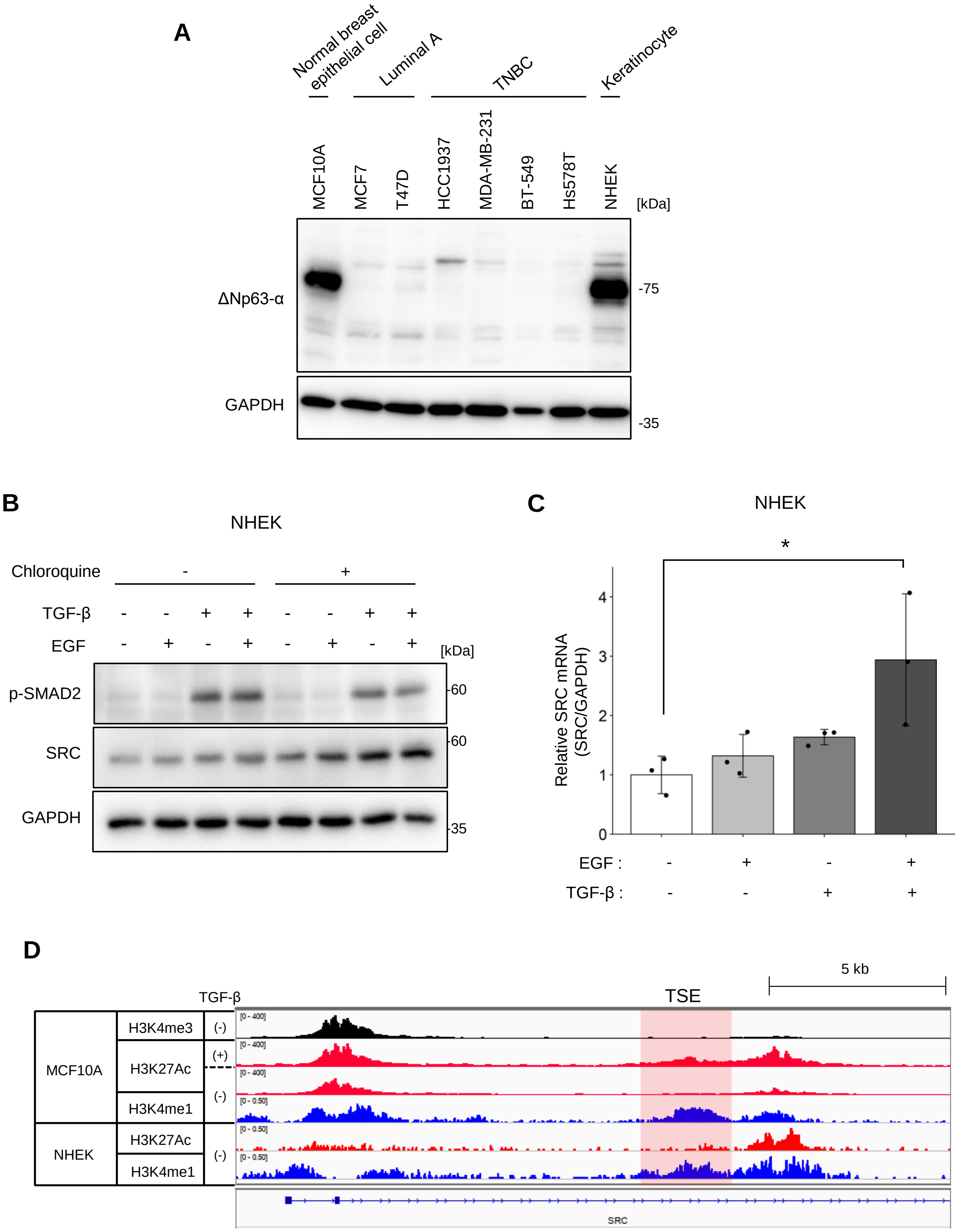

Fig. S7. Co-stimulation of TGF-β and EGF regulates SRC expression in p63-expressing normal human epidermal keratinocyte (NHEK).
(A) Immunoblot analysis for the expression of p63 in the indicated cell lines. TNBC, triple-negative breast cancer. (B) NHEK cells were treated with TGF-β1 (10 ng/ml) and/or EGF (20 ng/ml) for 24h. Chloroquine (100 μM) was added to the medium, and the cells were incubated for 2h before cell lysate collection. Cell lysates were subjected to immunoblotting using the indicated antibodies. (C) NHEK cells were treated with TGF-β1 (10 ng/ ml) and/or EGF (20 ng/ml) for 24h. Total RNA was isolated and subjected to quantitative real-time polymerase chain reaction PCR. (D) Genomic loci of *the SRC* promoter region in MCF10A and NHEK cells The IP targets are H3K4me3 (black), H3K27Ac (red), and H3K4me1 (blue). (C) Mean ratios ± SDs were obtained from three independent experiments. * $p < 0.05$; One-way ANOVA with Tukey's post hoc test.

Journal of Cell Science • Supplementary information

Blot transparency

1B

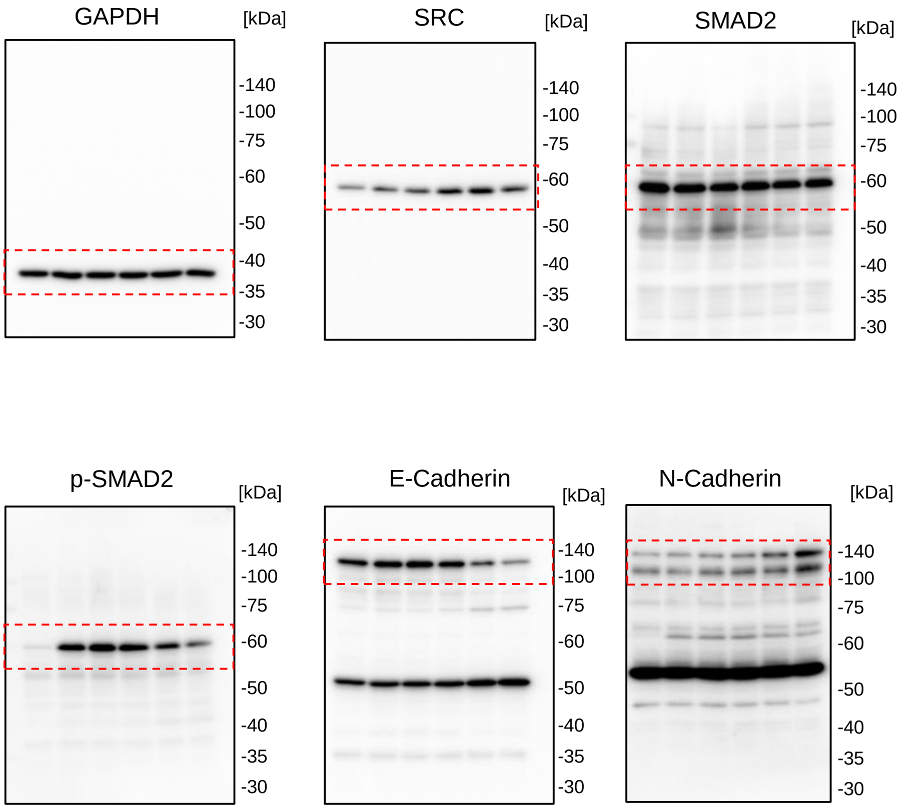

1G

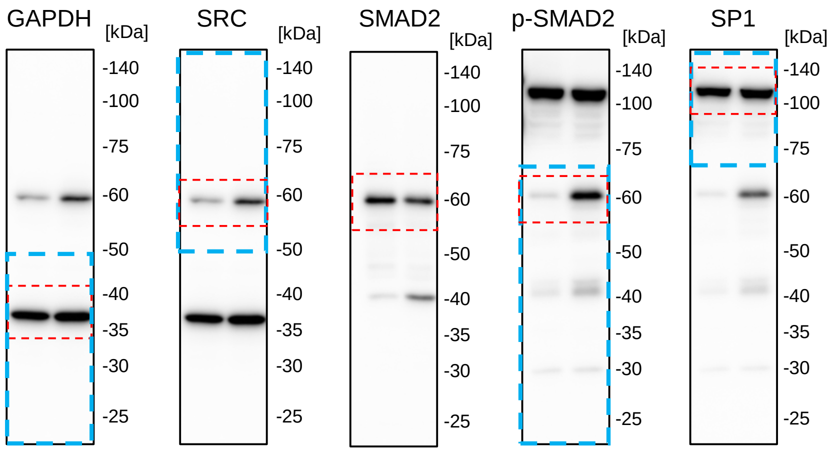

Fig. S8

(Continued)

Blot transparency

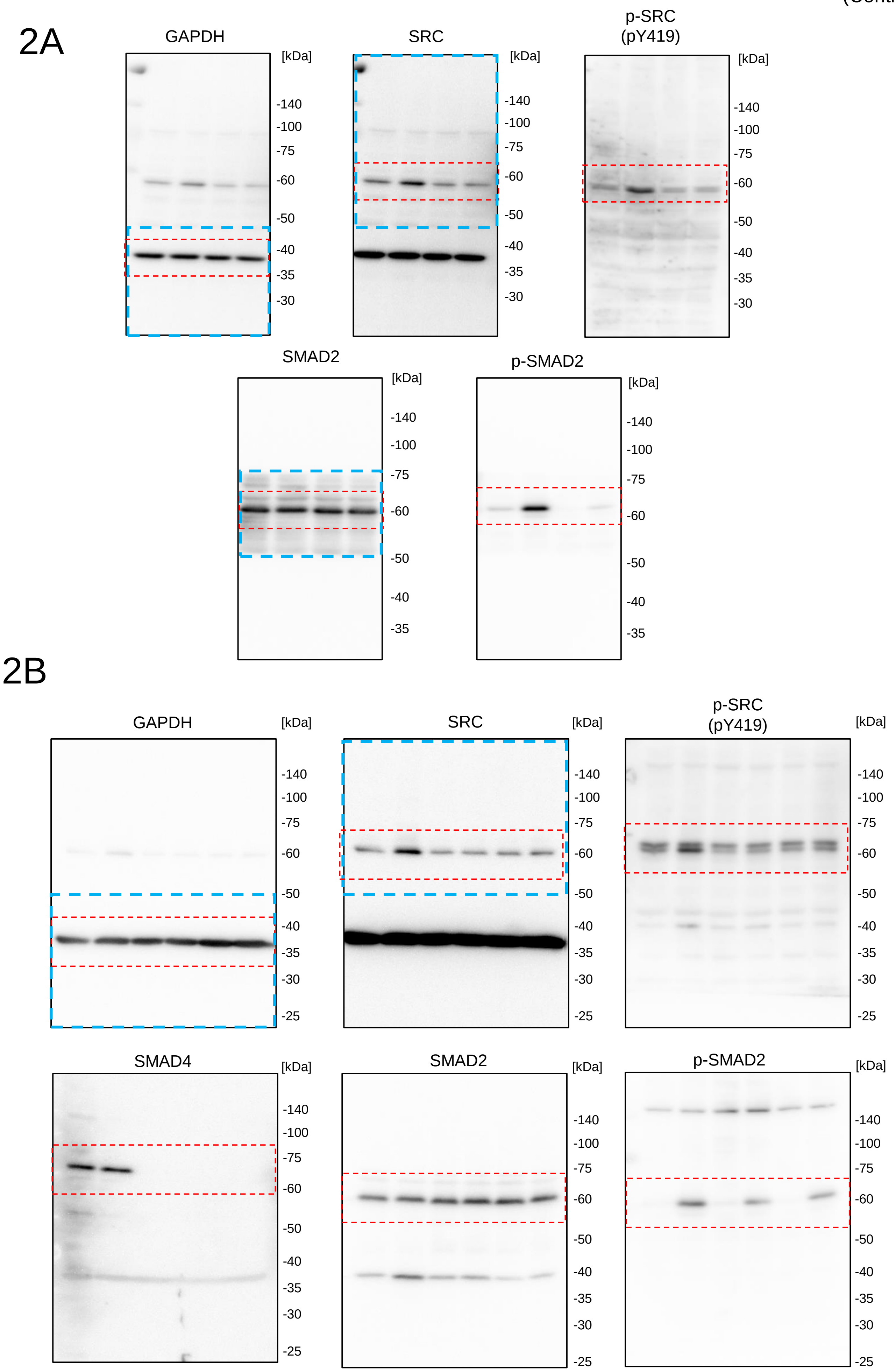

Journal of Cell Science • Supplementary information

Fig. S8

(Continued)

Blot transparency

4D

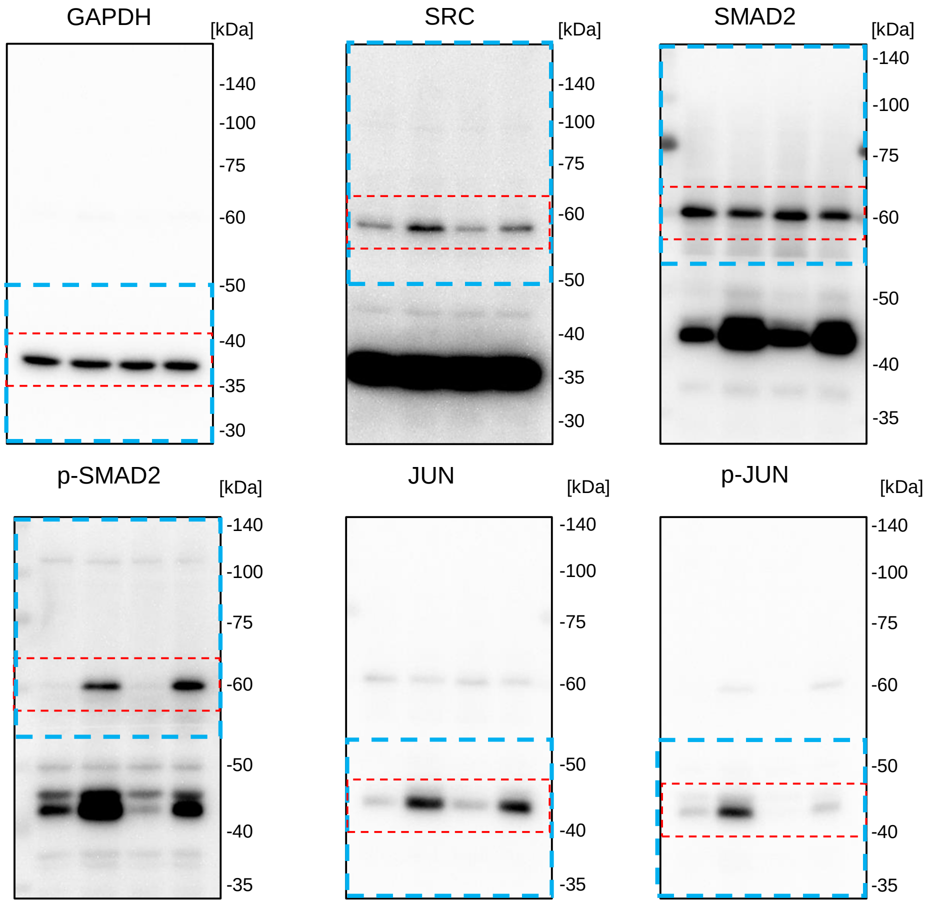

4F

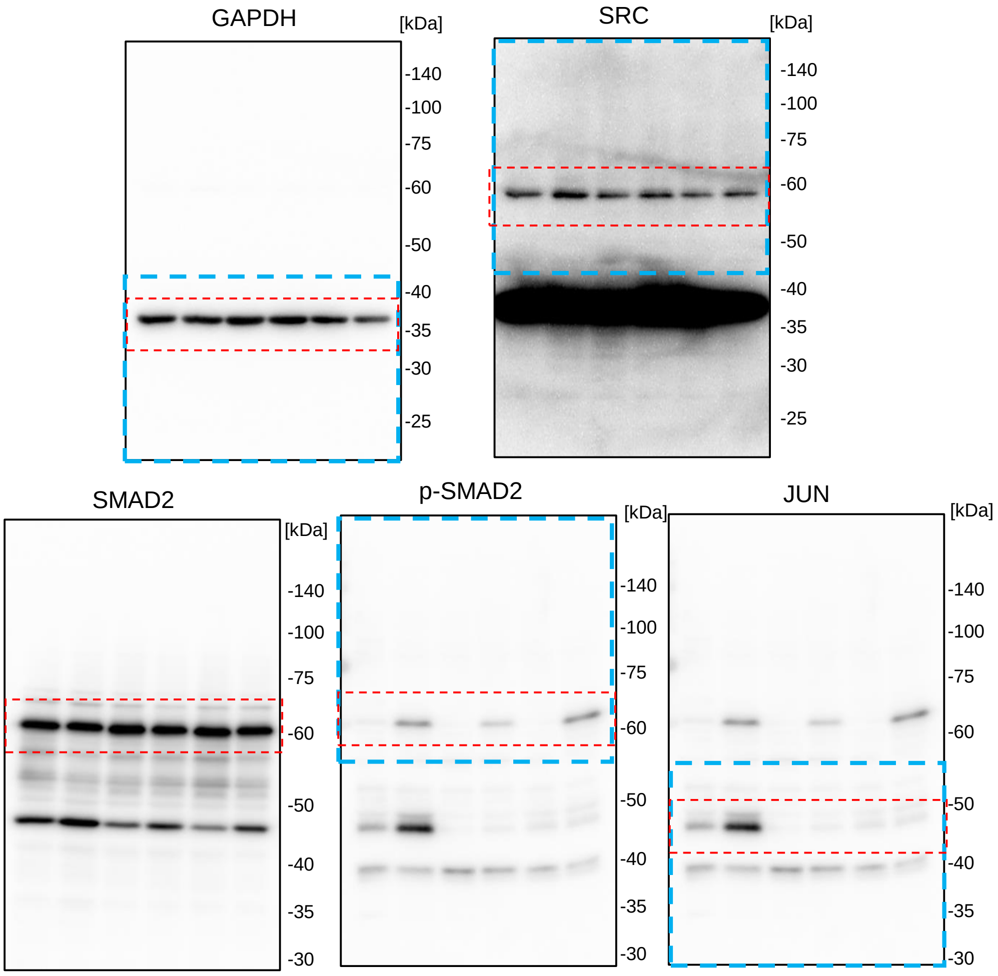

Journal of Cell Science • Supplementary information

Blot transparency

Fig. S8

(Continued)

5B

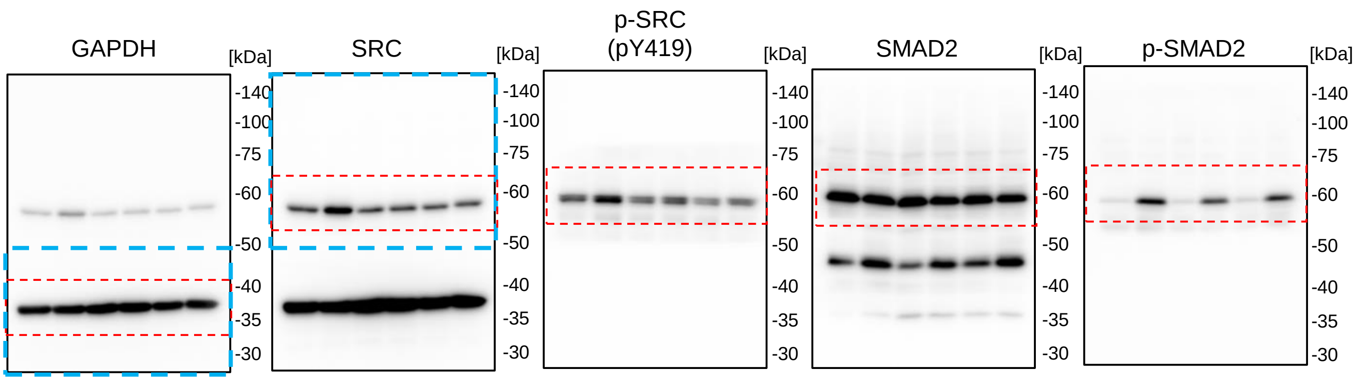

6A

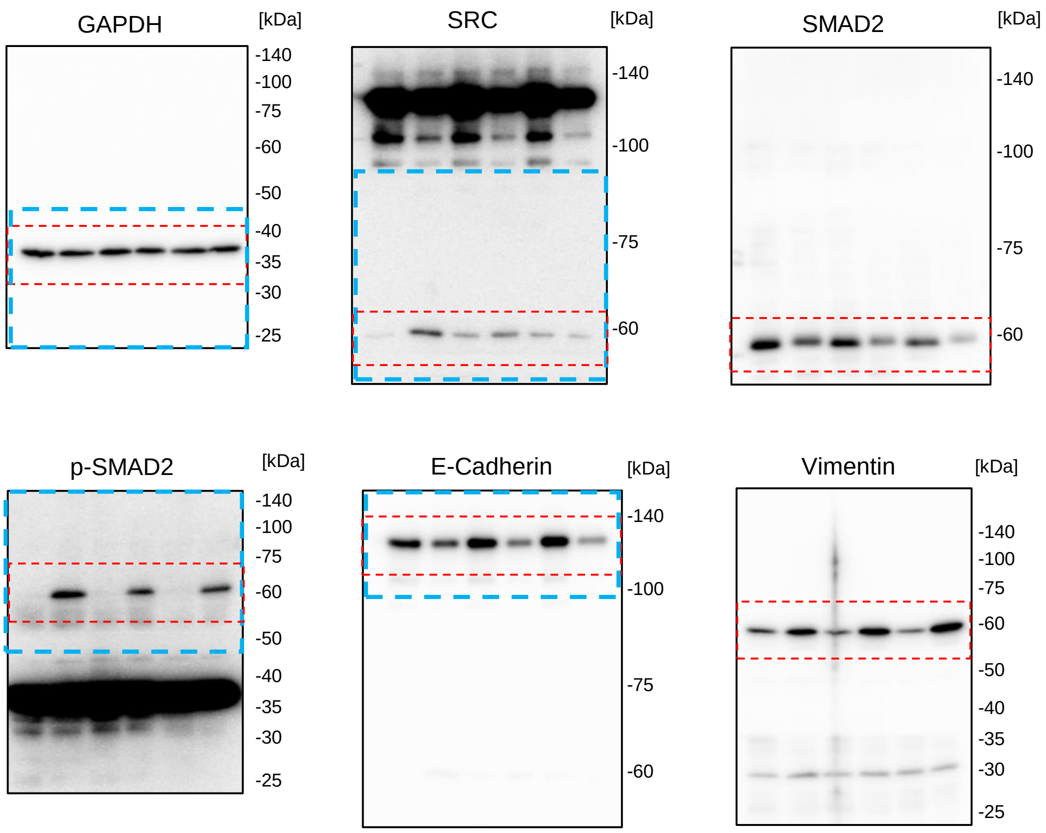

Journal of Cell Science • Supplementary information

Blot transparency

Fig. S8

(Continued)

6C

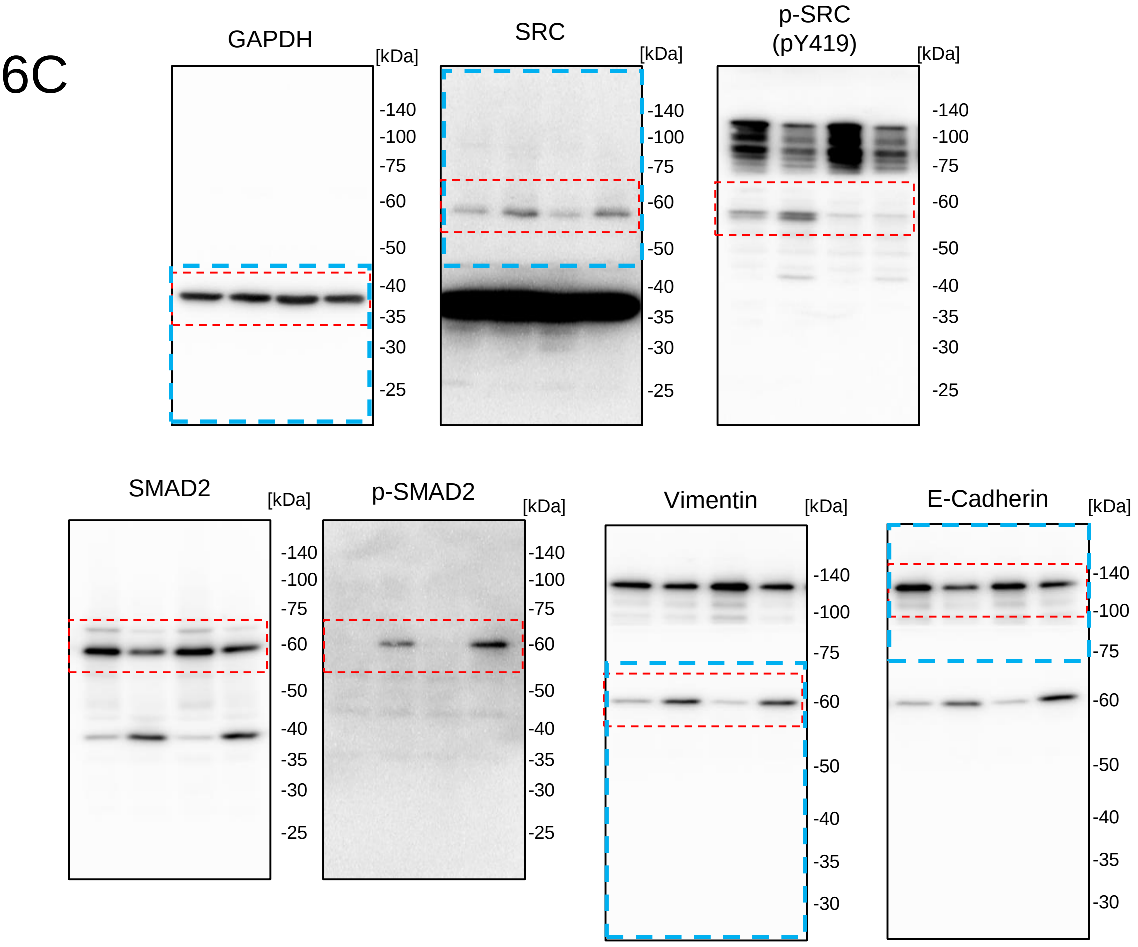

7A

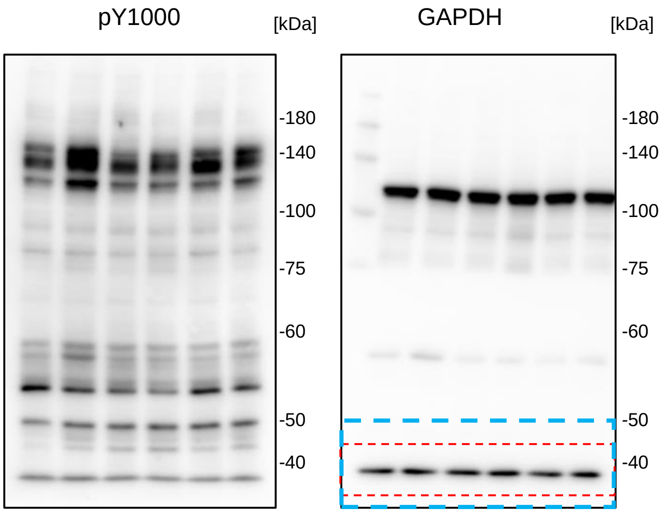

Journal of Cell Science • Supplementary information

Fig. S8

(Continued)

Blot transparency

7C

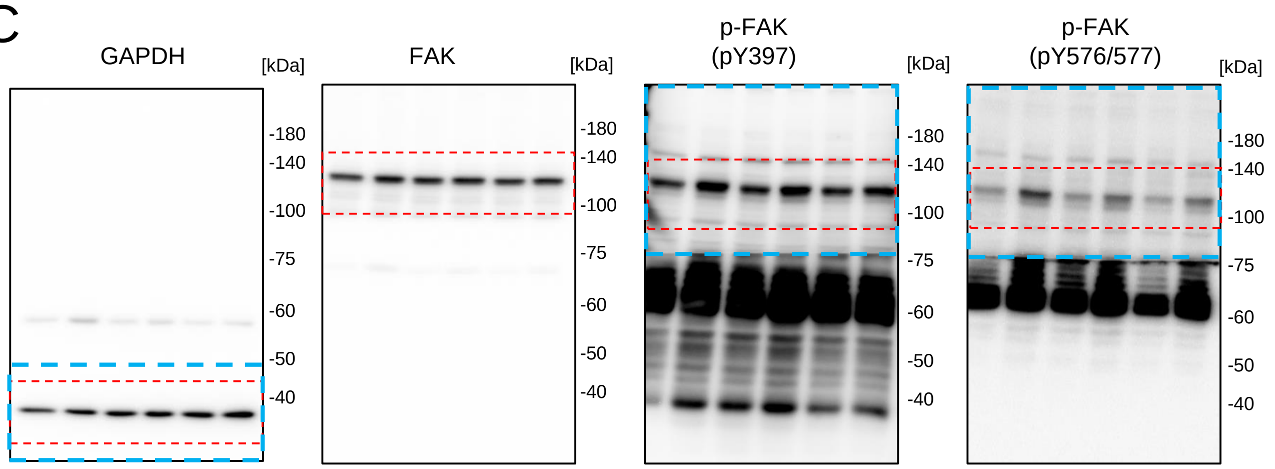

7E

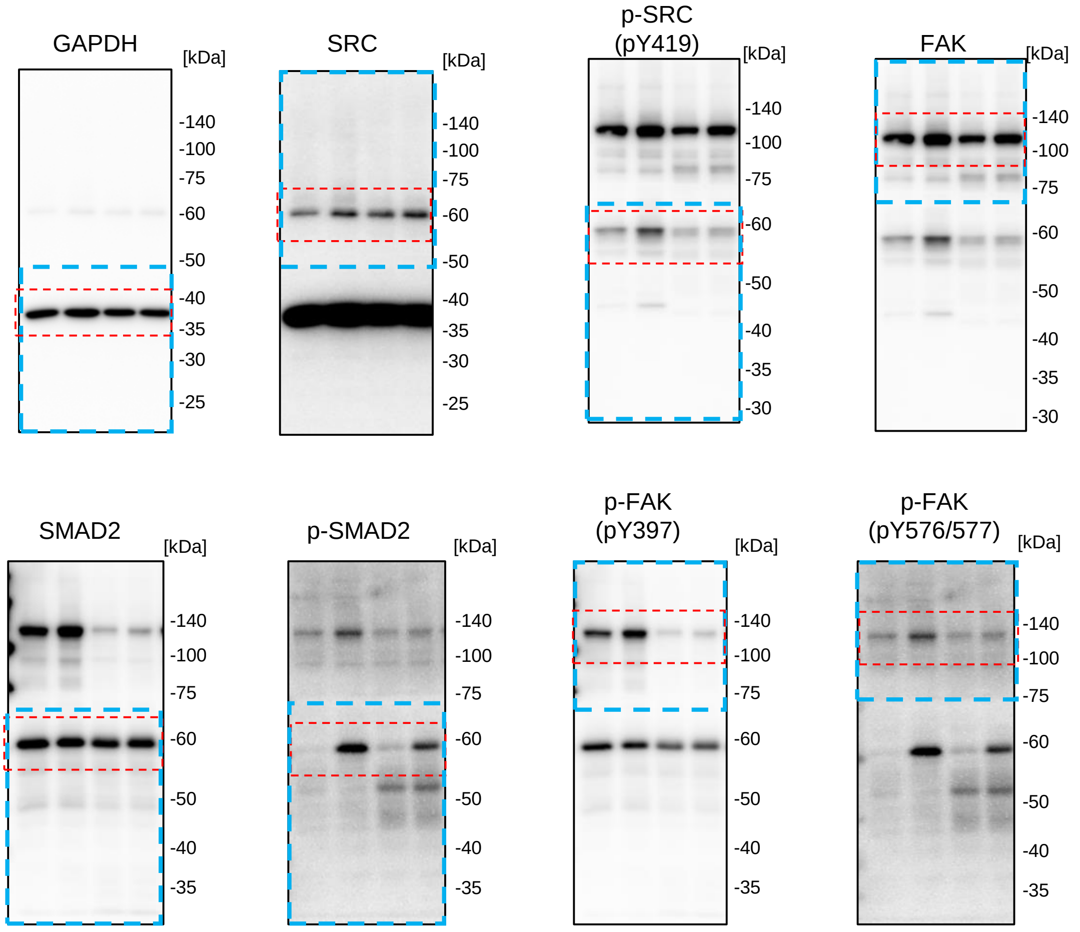

Fig. S8

(Continued)

Blot transparency

S1A

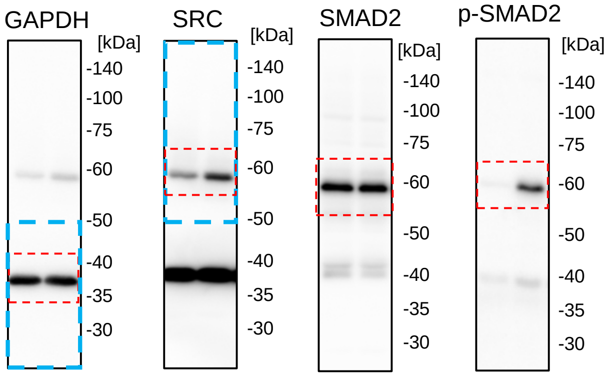

S4C

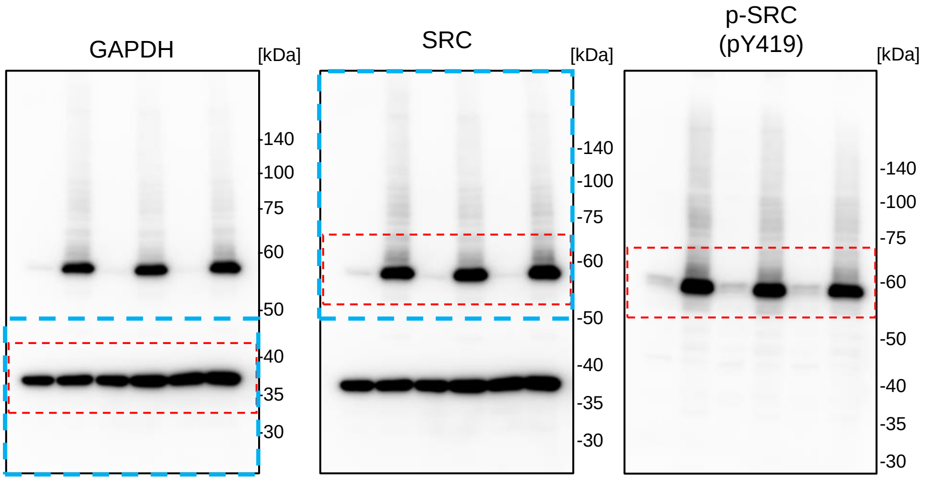

Blot transparency

Fig. S8

(Continued)

S5C

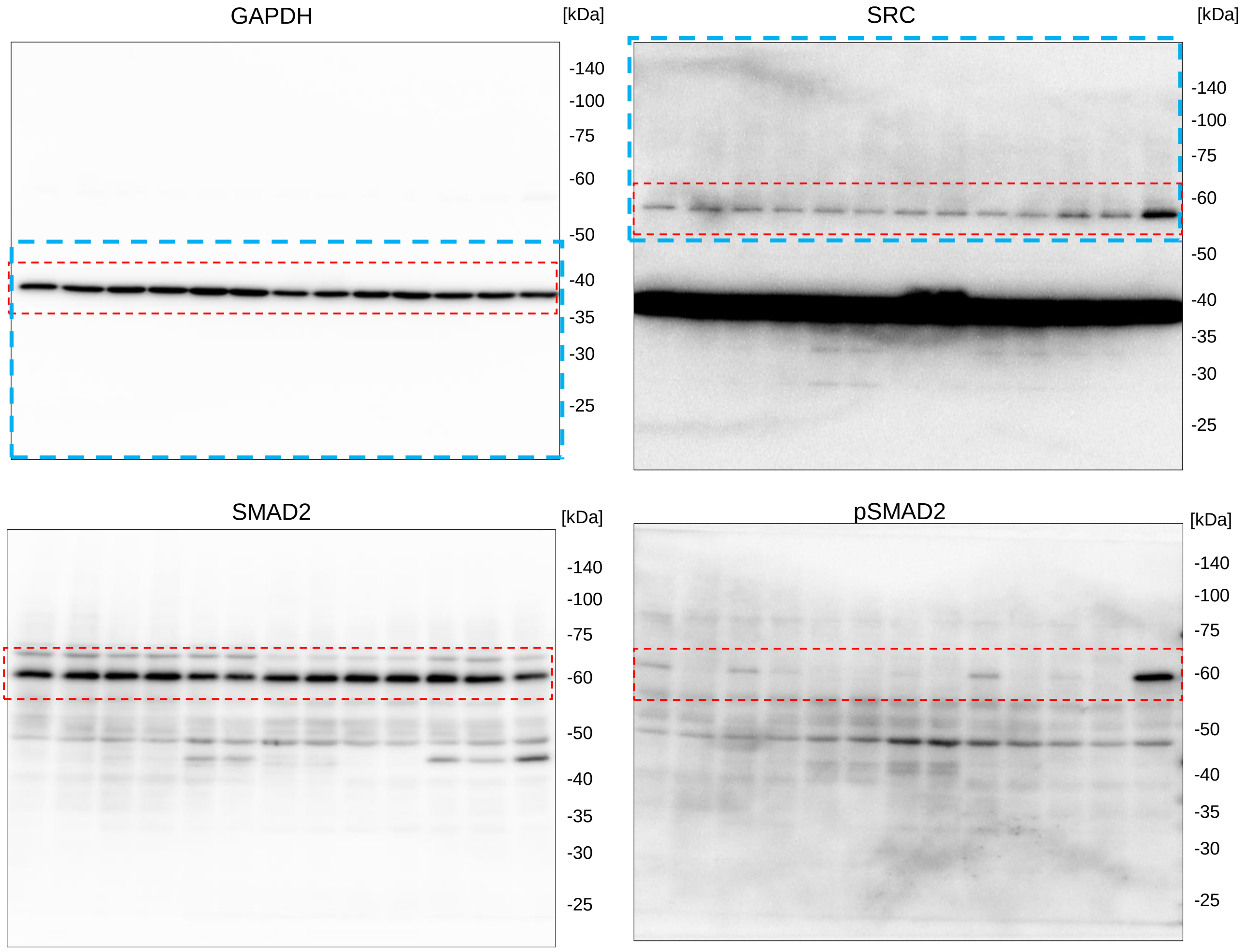

Journal of Cell Science • Supplementary information

Blot transparency

Fig. S8

(Continued)

S6A

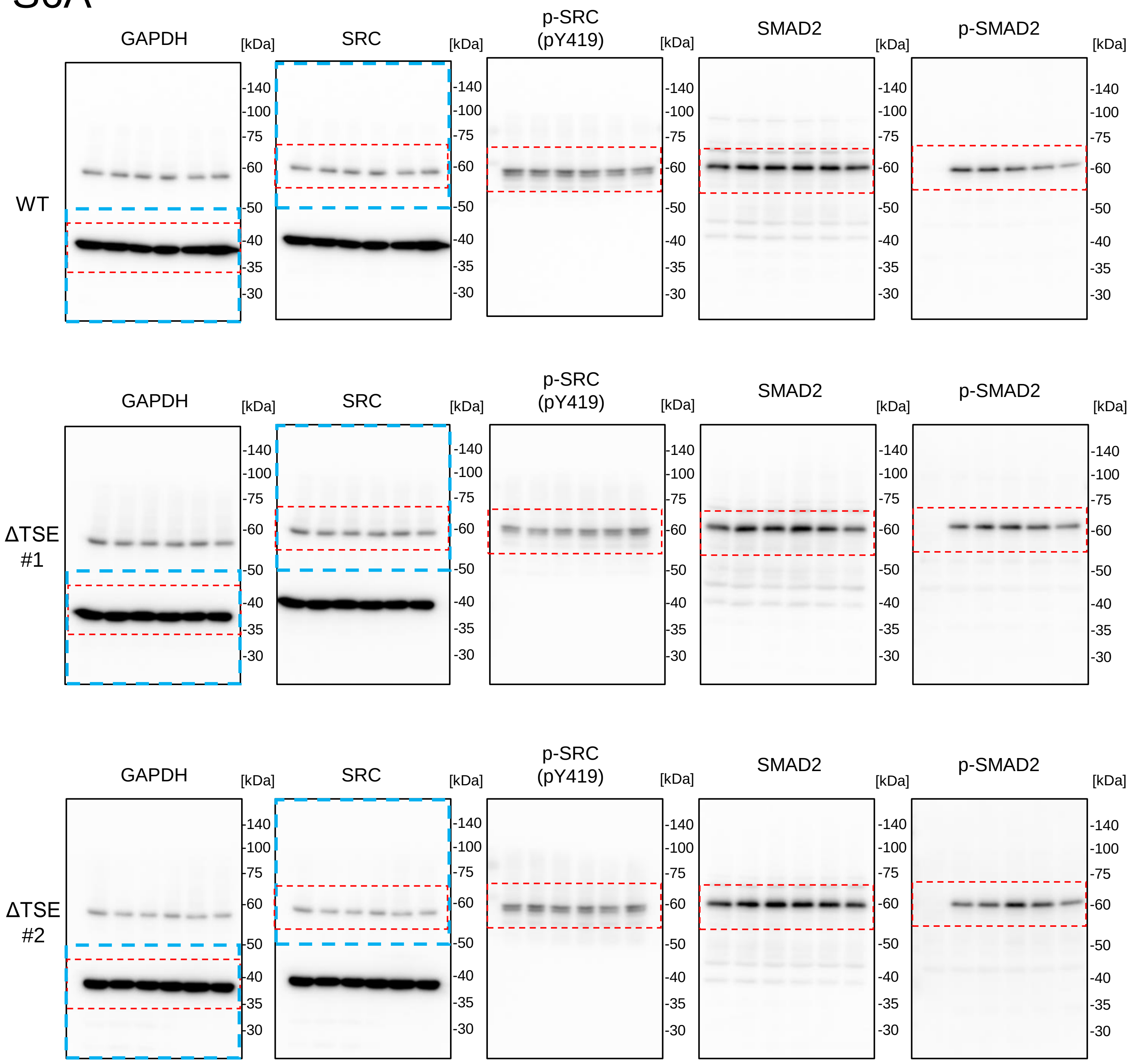

Journal of Cell Science • Supplementary information

Blot transparency

Fig. S8

(Continued)

S7A

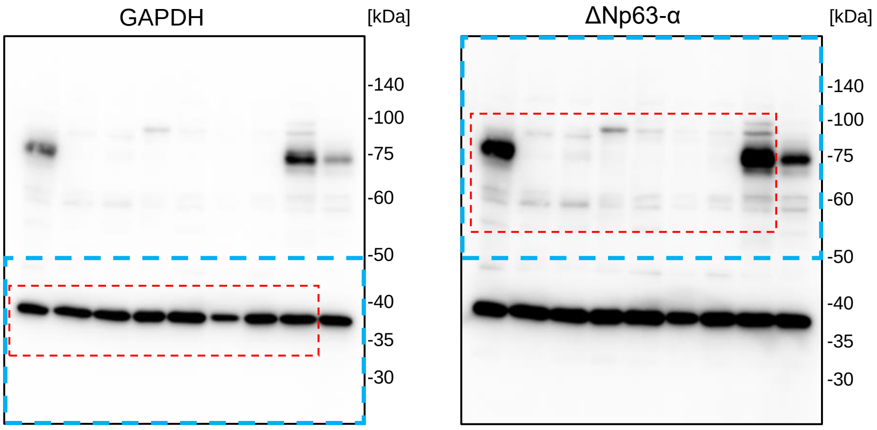

S7B

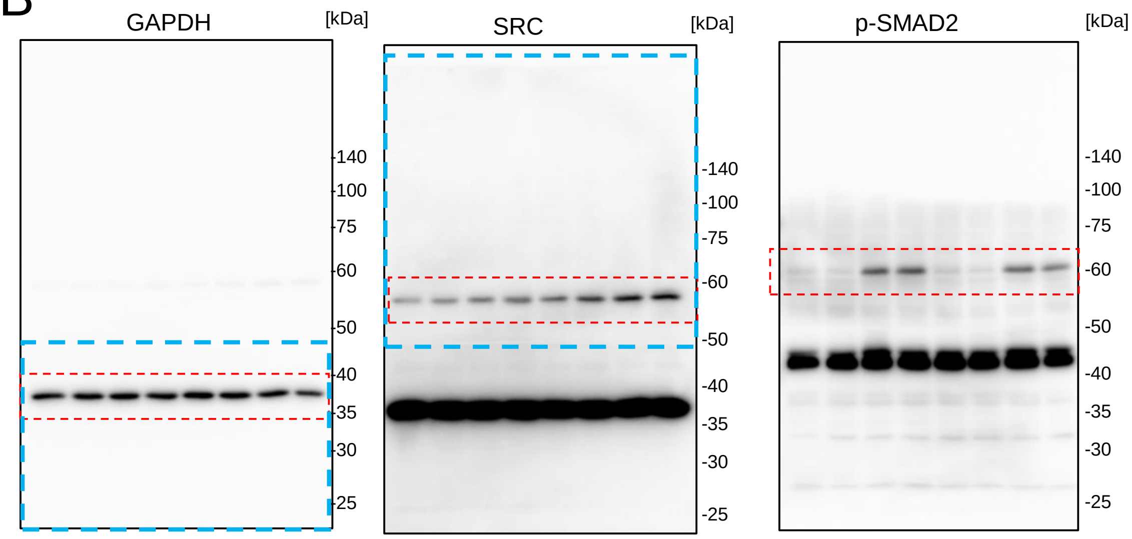

Fig. S8. Blot transparency

Journal of Cell Science • Supplementary information

Table S1. List of primers for vector constructions.

Cloning target		Sequence (5’ → 3’)
SRC 1A Promoter	Forward primer:	GTGAGGGGGCTGGGCTG
	Reverse primer:	CTGGGCCGGCGG
Enhancer. A	Forward primer:	GTTGTGGCAGGGAGGGGAG
	Reverse primer:	TACAAGAAAATGAGCCAGGCGTGG
Enhancer. B (TSE)	Forward primer:	AGTTGGCCATTTGCAGCGAG
	Reverse primer:	GCCACTTCCTCACAGAAAGCC
Enhancer. C	Forward primer:	GGCTTTCTGTGAGGAAGTGGC
	Reverse primer:	AGAACAGGTGTGGAGGAGGC
mTSE (SBS#1)	Forward primer:	ACGCCTGCTTGGCAGGAAGTGGTGGAGAC
	Reverse primer:	TCCTGCCAAGCAGGCGTCTTCGAGGAAAGG
ΔTSE (SBS#1)	Forward primer:	AAGACGCGGAAGTGGTGGAGACGTG
	Reverse primer:	CCACCACTTCCGCGTCTTCGAGGAAA
mTSE_1 (SBS#2)	Forward primer:	AGGAAGTGATGGAGACGTGTTGCAGCCC
	Reverse primer:	CGTCTCCATCACTTCCTGCCAGGCAGGCG
mTSE_2 (SBS#2)	Forward primer:	GGTGGAAACGTGTTGCAGCCCAAC
	Reverse primer:	CAACACGTTTCCACCACTTCCTGCC
ΔTSE (SBS#2)	Forward primer:	GGAAGTGGAGCCCAACTGGCAGATTC
	Reverse primer:	GTTGGGCTCCACTTCCTGCCAGGC
mTSE (AP-1)	Forward primer:	CGTGTTGCTGCCCAACTGGCAGATTCCC
	Reverse primer:	AGTTGGGCAGCAACACGTCTCCACCACTTC
Human SRC gene	Forward primer:	ATGGGTAGCAACAAGAGCAAGC
	Reverse primer:	CTAGAGGTTCTCCCCGGGCTGG

Table S2. List of siRNA.

Target gene		Sequence (5’ → 3’)
siJUN #1	Sense	GAUGGAAACGACCUUCUAU[dT][dT]
	Antisense	AUAGAAGGUCGUUUCCAUC[dT][dT]
siJUN #4	Sense	GGCCCUGAAGGAGGAGCCU[dT][dT]
	Antisense	AGGCUCCUCCUUCAGGGCC[dT][dG]

Journal of Cell Science • Supplementary information

Table S3. List of qPCR primers.

Target gene		Sequence (5' → 3')
SRC	Forward primer:	TCAACAACACAGAGGGAGAC
	Reverse primer:	CGTAGTTGCTGGGGATGTAG
GAPDH	Forward primer:	GCTCTCTGCTCCTCCTGTTC
	Reverse primer:	CGCCCAATACGACCAAATCC
JUN	Forward primer:	ACGACCTTCTATGACGATGCC
	Reverse primer:	CCAGGTTCAGGGTCATGCTC
(For Semi-qPCR in Fig. 1E)		
SRC Exon1A	Forward primer:	GTCTGCCCGTCCCGCTGGAC
SRC Exon1α	Forward primer:	AGCACAACCTGACCATCCTCACACTG
SRC Common	Forward primer:	GAAACCAGATGAGGACGCTGAGGCC
	Reverse primer:	AAAGGTGGTCACTCCACCGGCC
GAPDH	Forward primer:	CATCTTCCAGGAGCGAGATCCC
	Reverse primer:	TTCGTTGTCATACCAGGAAATGAGC
(For ChIP-qPCR in Fig. 4B)		
TSE	Forward primer:	CTGTCCATCCTTTCCTCGAA
	Reverse primer:	CTTGGGAATCTGCCAGTTG

Table S4. List of gRNA sequence and genotyping primers.

gRNA target		Sequence (5' → 3')
SMAD4		TCTGTCGATGCACGATTACT
Enhancer. B (TSE)		GACGCCTGCCTGGCAGGAAG
PCR target		
SMAD4 for genotyping	Forward primer:	GTTGCTGGAGGCTGTTGAAAC
	Reverse primer:	CCCCTCTCCCTCCTATGACAT
Enhancer. B for genotyping	Forward primer:	CTTCCTGGGGGAGGAGGTAC
	Reverse primer:	GCCACTTCCTCACAGAAAGCC

Journal of Cell Science • Supplementary information
