# Supplementary material for: A TGF-β-responsive enhancer regulates SRC expression and epithelial–mesenchymal transition-associated cell migration
Source: J Cell Sci. 2023 Aug 9;136(15):jcs261001. doi: 10.1242/jcs.261001 (PMC10445741; doi:10.1242/jcs.261001)
Supplement: Supplementary file 1 [file joces-136-261001_review_history.pdf]

A novel TGFß-responsive enhancer regulates SRC expression and epithelial-mesenchymal transition-associated cell migration

Soshi Noshita, Yuki Kubo, Kentaro Kajiwara, Daisuke Okuzaki, Shigeyuki Nada and Masato Okada

DOI: 10.1242/jcs.261001

Editor: Andrew Ewald

Review timeline
Original submission: 19 January 2023
Editorial decision: 22 February 2023
First revision received: 29 May 2023
Accepted: 30 June 2023

Original submission

First decision letter

MS ID#: JOCES/2023/261001

MS TITLE: A novel TGF-&[beta]-responsive enhancer regulates SRC expression and epithelial-mesenchymal transition-associated cell migration

AUTHORS: Soshi Noshita, Yuki Kubo, Kentaro Kajiwara, Daisuke Okuzaki, Shigeyuki Nada, and Masato Okada
ARTICLE TYPE: Research Article

We have now reached a decision on the above manuscript.

To see the reviewers' reports and a copy of this decision letter, please go to: https://submit-jcs.biologists.org and click on the 'Manuscripts with Decisions' queue in the Author Area. (Corresponding author only has access to reviews.)

As you will see, the reviewers share enthusiasm for the study but raise a number of substantial criticisms that prevent me from accepting the paper at this stage. They also highlight that a paper has been published recently on a similar topic (PMID: 36378749) and that it should be cited and discussed. They suggest, however, that a revised version might prove acceptable, if you can address their concerns. If you think that you can deal satisfactorily with the criticisms on revision, I would be pleased to see a revised manuscript. We would then return it to the reviewers.

Please ensure that you clearly highlight all changes made in the revised manuscript. Please avoid using 'Tracked changes' in Word files as these are lost in PDF conversion.

I should be grateful if you would also provide a point-by-point response detailing how you have dealt with all of the points raised by the reviewers in the 'Response to Reviewers' box. Please attend to all of the reviewers' comments. If you do not agree with any of their criticisms or suggestions please explain clearly why this is so.

© 2023. Published by The Company of Biologists under the terms of the Creative Commons Attribution License (https://creativecommons.org/licenses/by/4.0/).

<u>Reviewer 1</u>

Advance summary and potential significance to field

In the present study, the authors examined the role of SRC induction in TGF-ß-induced EMT. They identified an enhancer responsible for the induction of SRC by TGF-ß, and inactivated the enhancer by genome editing. Using the enhancer-inactivated cells, they showed that upregulation of SRC is essential for activation of FAK and promotion of cell motility in TGF-ß-stimulated cells. This is a nice strategy to unveil the role of SRC downstream of TGF-ß. However, a recent publication by Yakymovych et al. also reported the role of SRC in TGF-ß-induced FAK activation and cell motility (PMID: 36378749). Therefore, the novelty of this work has been, in part, diminished. In addition, Yakymovych et al. proposed a different mode of SRC activation by TGF-ß. Such discrepancy should be addressed to make a significant contribution to the field. Overall, presented data support the authors' conclusions.

Comments for the author

Major points:

1) One of the conclusions of this study is that TGF-ß activates the SRC pathway principally through upregulation of SRC proteins expression. Recently, however, TGF-ß type II receptor has been reported to promote autophosphorylation of SRC (PMID: 36378749). Because both works use the same cell line MCF10A, the paper should be cited and discussed. One important difference between two studies may be the different time points to assess SRC phosphorylation status. It is possible that direct activation of SCR by TßR complex plays a role in an early phase SRC activation, while upregulation of SRC protein is indispensable for a delayed phase SRC activation. I reccomend the authors to present data on SRC phosphorylation at the earlier time points in a dataset like Figure 5B.

2) Many cancer cells are stimulated by endogenous TGF-ß in an autocrine manner. Show if LY364979 affects the basal SRC expression in (breast) cancer cells with high levels of SRC expression. Such data would support the importance of findings reported in this paper.

3) Figure 2B and C (SMAD4-KO cells): Rescue experiments should be performed to verity that induction of SRC by TGF-ß requires SMAD4. Possiblity of any off-target effects should be excluded.

Minor points

4) Figure 6E: Y15 appears to inhibit Smad2 activation. Is this result reproducible? If so, it should be mentioned and discussed.

5) As for the role of SRC in FAK activation and cell motility, the previous report (PMID: 36378749) should be cited.

6) Page 4, line 2 from the bottom, the TGF-ß/SMAD pathway is essential for the regulation of SRC transcription: This is an overstatement, because SMAD4-independent SMAD signaling has been reported (for example, PMID: 26898331). In addition, SMAD4 also mediates BMP signaling and BMP ligands are often induced by TGF-ß stimulation. The statement here should be "SMAD4 is essential for the regulation of SRC transcription".

7) As for enrichment of the AP-1 motifs in SMAD binding regions, cite a paper by Koiuma et al. (PMID: 18955504).

8) Information on SMAD4 KO should be included in Experimental procedures.

9) Overall: SEs are not recommended. Use SDs.

10) ChIP-seq data should be deposited to the public database.

11) Figure S5, the Y-axis: Absorbance values higher than 2 are not reliable. Samples should be diluted and then subjected for measurement. If the authors did so, describe it in the legend. //

© 2023. Published by The Company of Biologists under the terms of the Creative Commons Attribution License (https://creativecommons.org/licenses/by/4.0/).

Reviewer 2

Advance summary and potential significance to field

Noshita et al. demonstrated that the Src transcript including exon 1A is upregulated by TGF-b/Smad and AP1, and the Src protein including exon1A contributes to TGF-b-mediated cell migration, but not cell growth. Their present study is recognized for novelty, whereas some critical results are missing in the present form. They must do their experiments to show further detailed molecular mechanism.

Comments for the author

1. (Fig. 2B) To avoid the off-target effect, they should use two independent Smad4-KO cells.

2. (Fig. 3 & 4) Although mutation analysis shows TSE is critical for TGF-b/Smad and AP1 to activate the Src gene including exon 1A, they have not shown any direct evidences that Smad and AP1 bind to TSE by gel shift assay, DNAP or ChIP assay. In addition, the evidence that Smad4 is included in Smad2/3/AP1 complex should be shown.

3. (Fig, 3C and D) canonical Smad-binding element (SBE) is AGAC or GTCT. One SBE in SBS#2 can be found, however they have not mutated this region. Why not?

4. (Fig. 4E) Like Question 1, try to do same experiments using different siRNA for Jun.

5. (Fig. 6F) When the Src protein including exon1A is put back to deltaTSE cells do the cells improve cell motility upon TGF-b stimulation? On the other hand, how about the Src protein including exon1alpha?

Reviewer 3

Advance summary and potential significance to field

In this manuscript, Noshita et al. reported their finding that TGFb regulates SRC expression through an intragenetic enhancer, termed TGFb-responsive SRC enhancer (TSE). They demonstrated that TSE knockout affected TGFb-induced migration but did not influence cell morphology. They hypothesized that the migration phenotype might be mediated by SRC recruitment of FAK. Overall, the connection between TGFb and SRC expression is interesting. The discovery of TSE is novel. The downstream impact of this pathway on cell migration but not cell morphology could be relevant for our understanding of metastasis. However, some of the key aspects need to be strengthened to support the overall hypothesis. There is a missed opportunity to increase the manuscript's impact.

1. The epistasis between TGFb and SRC is not fully established.
Although TSE knockout abolished TGFb's effects on cell migration independent approaches such as knockdown of SRC expression or inhibition of SRC kinase activities in the presence of TGFb will be required to rule out potential off-target effects of CRISPR-Cas9 mediated knockout.

2. Similarly, although SRC/FAK interaction and their roles in cell migration have been well documented, it would be important to establish that FAK is indeed responsible for the observed migration phenotype in this particular system.

3. According to the authors, SRC activation represents a downstream effect of TGFb that separates EMT markers (e.g., E-cadherin) from migration.
This is potentially very interesting and should be further investigated.
Therefore, the authors are highly encouraged to further the studies along this direction. For instance, will TSE knockout result in more "collective migration" of cell clusters in a 3D system?

© 2023. Published by The Company of Biologists under the terms of the Creative Commons Attribution License (https://creativecommons.org/licenses/by/4.0/).

Comments for the author

Suggestions are provided together with the comments.

First revision

Author response to reviewers' comments

MS ID#: JOCES/2023/261001

A TGF-β-responsive enhancer regulates SRC expression and epithelial-mesenchymal transition-associated cell migration
Soshi Noshita, Yuki Kubo, Kentaro Kajiwara, Daisuke Okuzaki, Shigeyuki Nada, and Masato Okada

We thank all the reviewers for taking the time to review our manuscript and for providing thoughtful comments. In this revision, we performed additional experiments and modified the manuscript to address all the comments raised by the reviewers. These data have now been included in the revised manuscript (please refer to the new figures if we mention the results in the response letter).

Reviewer 1 Advance Summary and Potential Significance to Field:

In the present study, the authors examined the role of SRC induction in TGF-β-induced EMT. They identified an enhancer responsible for the induction of SRC by TGF-β, and inactivated the enhancer by genome editing. Using the enhancer-inactivated cells, they showed that upregulation of SRC is essential for activation of FAK and promotion of cell motility in TGF-β-stimulated cells. This is a nice strategy to unveil the role of SRC downstream of TGF-β. However, a recent publication by Yakymovych et al. also reported the role of SRC in TGF-β-induced FAK activation and cell motility (PMID: 36378749). Therefore, the novelty of this work has been, in part, diminished. In addition, Yakymovych et al. proposed a different mode of SRC activation by TGF-β. Such discrepancy should be addressed to make a significant contribution to the field. Overall, presented data support the authors' conclusions.

Reviewer 1 Comments for the Author:
Major points:
1)One of the conclusions of this study is that TGF-β activates the SRC pathway principally through upregulation of SRC proteins expression. Recently, however, TGF-β type II receptor has been reported to promote autophosphorylation of SRC (PMID: 36378749). Because both works use the same cell line, MCF10A, the paper should be cited and discussed. One important difference between two studies may be the different time points to assess SRC phosphorylation status. It is possible that direct activation of SCR by TβR complex plays a role in an early phase SRC activation, while upregulation of SRC protein is indispensable for a delayed phase SRC activation. I reccomend the authors to present data on SRC phosphorylation at the earlier time points in a dataset like Figure 5B.

Response:

We appreciate the thoughtful comments and the opportunity to clarify these important points. As the reviewer has pointed out, the time point is one of the major differences between the recent paper by Yakymovych et al. and our present study. Notably, immunoblotting data for fibronectin, PAI-1, and p- Smad2 are shown using MCF10A cells; however, immunoblotting data for SRC and p-SRC in MCF10A cells were not included in a previous paper. Hence, we strongly agree with the reviewer's suggestion that we need to present data on SRC phosphorylation at earlier time points in wild-type and TSE-mutant MCF10A cells.

We have performed short-time TGF-β stimulation in MCF10A referring to the experimental procedure

© 2023. Published by The Company of Biologists under the terms of the Creative Commons Attribution License (https://creativecommons.org/licenses/by/4.0/).

reported by Yakymovych et al., and the results are shown in Fig S6. A previous study showed that TGF-β stimulation in HEK293T, MEF, and PC3U cells resulted in 5- to 10-fold activation of SRC with a peak at 30 min (Fig 1A, C and S1A in a previous study by Yakymovych et al.), however, our results showed that SRC was not significantly activated by short-term TGF-β stimulation in both wild- type and TSE-mutant MCF10A cells (Fig S6A and B).

Notably, Yakymovych et al. mentioned in the discussion part of their study that whether TGF-β-induced SRC expression and the SRC-activating mechanism shown by them can occur in parallel remains to be determined, and we consider that our findings can provide important implications for this question. We showed that the total amount of active SRC decreased in TSE-mutant MCF10A cells (Fig 5B). This suggests that the increase in the total amount of active SRC induced by stimulation is mainly attributable to TSE-mediated SRC expression. In addition, SRC activation was not detected by short-time TGF-β stimulation in MCF10A (Fig S6). Taken together, these results suggest that other mechanisms activate SRC, at least in MCF10A cells. Importantly, this hypothesis is supported by data from a previous study by Yakymovych et al. According to their study, TGF-β-induced SRC activation is independent of the activity of TβRI in HEK293T and PC3U (Fig 1A, D and S1A in previous study by Yakymovych et al.), and consistently, the TβRI inhibitor SB505124 failed to inhibit EMT-induced cell motility in PC3U cells (Fig S5A in a previous study by Yakymovych et al.). However, they also described that the TβRI inhibitor inhibited TGF-β-induced cell migration in MCF10A, and we also obtained the same result using the TβRI inhibitor in MCF10A (Fig S4). These results are inconsistent with the statement that EMT-induced SRC activation is not dependent on the kinase activity of the TβRI.

Our study showed that the TGF-β/SMAD signalling pathway, which is dependent on TβRI activity, is essential for TSE-mediated SRC expression and subsequent upregulation of cell motility by activating SRC/FAK circuit in MCF10A. Based on our hypothesis that there are other mechanisms underlying SRC activation in MCF10A cells, this discrepancy presented in the previous report can be explained and solved by the findings presented in this paper.

In conclusion, we again insist that the TGF-β/SMAD signalling pathway and following TSE- mediated SRC expression, rather than the type II TGF-β receptor-dependent SRC activation, are essential for TGF-β-induced cell motility at least in MCF10A. In addition, we emphasise that the novelties of our study are the identification of TSE and the finding that TSE-mediated SRC expression is essential for TGF-β-induced cell motility. Yakymovych et al. did not interfere with these novelties. We appreciate the opportunity to clarify this point and have included this in the Discussion section of the revised manuscript. (page 10, para 1 and page11, para 3)

2) Many cancer cells are stimulated by endogenous TGF-β in an autocrine manner. Show if LY364979 affects the basal SRC expression in (breast) cancer cells with high levels of SRC expression. Such data would support the importance of findings reported in this paper.
Response:

We appreciate the reviewer's insightful suggestion. To address whether endogenous autocrine TGF-β affects the basal SRC expression, we have performed LY364947 treatment in several kinds of breast cancer cell lines, and this result was added to Fig S5C. In the absence of LY364947, SMAD2 phosphorylation was detected in MCF7, T47D, BT-549, Hs578T, and MCF10A cells, implying that autocrine TGF-β signalling is induced in the basal state of these cell lines. In the presence of LY364947, SMAD2 phosphorylation was suppressed; however, the basal expression of SRC did not change significantly. Notably, TCGA analysis revealed that SRC was, on average, 1.8-fold overexpressed in patient-derived tissues (Fig S5B); however, SRC was not overexpressed in these breast cancer cell lines compared to the normal breast epithelial cell line MCF10A (Fig S5C). Based on these results, we believe that further *in vivo* investigations are needed to understand the mechanisms by which SRC is overexpressed in human cancer tissues, and our findings can contribute to this elucidation. We appreciate the opportunity to clarify this point and have included it in the Discussion section of the revised manuscript. (page 11, para 2)

3)Figure 2B and C (SMAD4-KO cells): Rescue experiments should be performed to verity that induction of SRC by TGF-β requires SMAD4. Possiblity of any off-target effects should be excluded.

© 2023. Published by The Company of Biologists under the terms of the Creative Commons Attribution License (https://creativecommons.org/licenses/by/4.0/).

Response:
Thank you for this helpful suggestion. As pointed out, concerns regarding the possibility of off-target effects should be excluded; Reviewer 2 also mentioned this point. To exclude the possibility of off- target effects, additional experiments were performed using two independent SMAD4-KO clones. In both SMAD4-KO clones, TGF-β-induced SRC expression was suppressed at both protein and mRNA levels, suggesting that SMAD4 is essential for TGF-β-induced SRC expression. These data are now included in Figures 2B and C.

Minor points

4) Figure 6E: Y15 appears to inhibit Smad2 activation. Is this result reproducible? If so, it should be mentioned and discussed.

Response:

Thank you for this thoughtful comment. First, we made a mistake with the concentration of the Y15 written in the legend of Fig. 7 and have now corrected it (5 uM → 10 uM). This change did not affect the original claims. In advance, we have confirmed the effect of the dose of Y15 on the FAK phosphorylation and the result is shown below. In MCF10A, more than 10 uM of Y15 can inhibit the autophosphorylation of FAK (pY397). As pointed out by the reviewer, the SMAD2 phosphorylation is partially decreased at 10 uM of Y15 and excessive concentration abolished SMAD2 phosphorylation. To reduce the side effect of SMAD2 phosphorylation, we used Y15 at the concentration of 10 uM in

NOTE: We have removed unpublished data that had been provided for the referees in confidence.

the present study. Importantly, previous studies have also reported that TGF-β-induced SMAD phosphorylation is attenuated by inhibition of FAK by another type of FAK inhibitor (Ding et al., 2017) or knockdown by siRNA (Park et al., 2010). These results imply crosstalk between TGF-β signalling and FAK; however, the molecular mechanisms underlying this crosstalk remains unknown. As suggested, we have included this information in the revised version of the manuscript. (page 9, para 2)

5)As for the role of SRC in FAK activation and cell motility, the previous report (PMID: 36378749) should be cited.

Response:
We agree with the reviewer's suggestion and have cited this report accordingly. (page 10, para 1)

6)Page 4, line 2 from the bottom, the TGF-β/SMAD pathway is essential for the regulation of SRC transcription: This is an overstatement, because SMAD4-independent SMAD signaling has been reported (for example, PMID: 26898331). In addition, SMAD4 also mediates BMP signaling and BMP ligands are often induced by TGF-β stimulation. The statement here should be "SMAD4 is essential for the regulation of SRC transcription".

Response:

We appreciate the Reviewer's thoughtful comments. We focused on the canonical TGF-β/SMAD signalling pathway mediated by the heterotrimeric complex of phosphorylated SMAD2/3 and SMAD4. But, as pointed out by the reviewer, SMAD4 KO does not mean the defect of the whole TGF-β/SMAD signalling pathway, so we agree with the reviewer's comment. To improve the accuracy of the statement, we rephrased it. (page 5, para 3)

7)As for enrichment of the AP-1 motifs in SMAD binding regions, cite a paper by Koiuma et al. (PMID: 18955504).
Response:
Thank you for this helpful suggestion. We have cited this report as follows: (page 7, para 1)

8)Information on SMAD4 KO should be included in Experimental procedures.

© 2023. Published by The Company of Biologists under the terms of the Creative Commons Attribution License
(https://creativecommons.org/licenses/by/4.0/).

Response:
We thank the reviewer for the careful review and have now included the sequence information of the gRNA and sequence primers used for SMAD4 knockout in Supplementary Table S4.

9) Overall: SEs are not recommended. Use SDs.
Response:
We have modified all graphs using SDs.

10) ChIP-seq data should be deposited to the public database.
Response:
We submitted the ChIP-seq data under the Gene Expression Omnibus (GEO) accession number GSE216432.

11)Figure S5, the Y-axis: Absorbance values higher than 2 are not reliable. Samples should be diluted and then subjected for measurement. If the authors did so, describe it in the legend.
Response:

Thank you for this helpful suggestion. To avoid excessive absorbance values, we modified the proliferation assay to shorten the reaction time by 1 h after the WST-8 addition. Using this method, we performed a proliferation assay, and the absorbance values did not exceed 2 at any time point. These data do not affect the original claims. We consider this result to be more reliable and have replaced the graph with new data in the revised manuscript (Fig S3).

Reviewer 2 Advance Summary and Potential Significance to Field:

Noshita et al. demonstrated that the Src transcript including exon 1A is upregulated by TGF-b/Smad and AP1, and the Src protein including exon1A contributes to TGF-b-mediated cell migration, but not cell growth. Their present study is recognized for novelty, whereas some critical results are missing in the present form. They must do their experiments to show further detailed molecular mechanism.

Reviewer 2 Comments for the Author:

1. (Fig. 2B) To avoid the off-target effect, they should use two independent Smad4-KO cells.
Response:
Thank you for this helpful suggestion. As suggested, we have performed additional experiments using two independent SMAD4-KO clones. In both SMAD4-KO clones, TGF-ß-induced SRC expression was suppressed at both protein and mRNA levels, suggesting that SMAD4 is essential for TGF-ß-induced SRC expression. These data are now included in Figures 2B and C.

2. (Fig. 3 & 4) Although mutation analysis shows TSE is critical for TGF-b/Smad and AP1 to activate the Src gene including exon 1A, they have not shown any direct evidences that Smad and AP1 bind to TSE by gel shift assay, DNAP or ChIP assay. In addition, the evidence that Smad4 is included in Smad2/3/AP1 complex should be shown.
Response:

Thank you for your valuable comments. We performed a ChIP-qPCR assay using monoclonal antibodies targeting SMAD2, SMAD3, SMAD4, and JUN. Similar to the ChIP-Seq results shown in Fig 3A and 4A, the ChIP-qPCR results showed that TSE was enriched using anti-SMAD2, anti- SMAD3, and anti-JUN antibodies. The enrichment was detected using an anti-SMAD4 antibody. Taken together, these results suggest that the SMAD2/3/4 complex and AP-1 are recruited at the TSE region in the presence of TGF-ß. These data have been added to Fig 4B in the revised manuscript. The antibodies and primers used in this experiment are now included in the Experimental Procedures section and Supplementary Table S3, respectively. (page 7, para 1)

3. (Fig, 3C and D) canonical Smad-binding element (SBE) is AGAC or GTCT. One SBE in SBS#2 can be found, however they have not mutated this region. Why not?

© 2023. Published by The Company of Biologists under the terms of the Creative Commons Attribution License (https://creativecommons.org/licenses/by/4.0/).

Response:

We appreciate the Reviewer's thoughtful comments. At that point, we conducted motif enrichment analysis using the JASPER database to find transcription factor candidates that have been reported to be related to TGF-ß signalling and have been also predicted to bind to enhancer B. Through these analyses, we have found that the transcription factor CREB is one of the candidates. Previous studies showed that CREB cooperates with SMAD3 in the presence of TGF-ß and regulates the TGF-ß- induced gene expression (Liu et al., 2006; Shen et al., 1998), with the CREB-binding motif (ACGT) also present in SBS#2 (Fig 3C). At that time, we could not exclude the possibility of a contribution from CREB. To introduce a 1bp substitution which can affect SMAD binding but not CREB binding,

we avoided introducing the mutation to the canonical Smad-binding element present in enhancer B. However, as pointed out by the reviewer, we recognised the importance of estimating the effect of the mutation introduced in this Smad-binding element. Hence, we created another luciferase reporter vector which possesses a 1bp substitution at the SBE in SBS#2 (Fig 3C) and performed a luciferase reporter assay to confirm the enhancer activity. Importantly, TGF-ß-induced *SRC* promoter activity decreased at the same level as that of the "P-ΔB (SBS#2)", indicating that canonical SBE in SBS#2 is essential for the enhancer activity. The results are now included in Fig 3D of the revised manuscript, and primer information is included in Supplementary Table S1.

4. (Fig. 4E) Like Question 1, try to do same experiments using different siRNA for Jun.

Response:

As suggested, we have performed the same experiments using two siRNAs, each targeting a different JUN mRNA sequence. The results are shown in Fig 4F and G. The expression of JUN protein was downregulated in the presence or absence of TGF-ß, and TGF-ß-induced SRC expression was reduced by JUN knockdown. We have also included information on the newly obtained siRNAs (Supplementary Table S2).

5. (Fig. 6F) When the Src protein including exon1A is put back to deltaTSE cells, do the cells improve cell motility upon TGF-b stimulation? On the other hand, how about the Src protein including exon1alpha?

Response:

Thank you for your valuable comments. As pointed out by the reviewer, there are two types of SRC transcripts, one containing exon1A and the other containing exon1α. Importantly, however, both exons are included in 5'-UTR regions of each mature SRC mRNA, and the remaining sequences of both variants are common. These transcriptional variants encoded identical SRC proteins. To clarify this point, we added text (page 5, para 2) and modified the schematic (Fig 1E).

We agree that it is important to examine whether EMT-induced cell motility can be restored by SRC protein overexpression in TSE cell lines. To bypass TSE-mediated SRC expression, we established cell lines which stably overexpressed wild-type SRC protein (Fig S4C) and performed a wound healing assay (Fig S4D and E). The results showed that TGF-ß-induced cell motility was recovered by overexpression of wild-type SRC protein in ΔTSE cell lines, suggesting that overexpression of SRC protein can upregulates cell motility. Notably, basal motility was enhanced by SRC overexpression, implying that fine-tuning SRC expression at the endogenous level is important for regulating cell motility in the basal state. Therefore, it is likely that TGF-ß regulates both TSE- mediated SRC expression and cell motility. We have included these data and the discussion above in the revised manuscript. (page 10, para 2)

Reviewer 3 Advance Summary and Potential Significance to Field:

In this manuscript, Noshita et al. reported their finding that TGFb regulates SRC expression through an intragenetic enhancer, termed TGFb-responsive SRC enhancer (TSE). They demonstrated that TSE knockout affected TGFb-induced migration but did not influence cell morphology. They hypothesized that the migration phenotype might be mediated by SRC recruitment of FAK. Overall, the connection between TGFb and SRC expression is interesting. The discovery of TSE is novel. The downstream impact of this pathway on cell migration but not cell morphology could be relevant for our understanding of metastasis. However, some of the key aspects need to be strengthened to support the overall hypothesis. There is a

© 2023. Published by The Company of Biologists under the terms of the Creative Commons Attribution License (https://creativecommons.org/licenses/by/4.0/).

missed opportunity to incre ase the manuscript’s impact.

1.The epistasis between TGFb and SRC is not fully established. Although TSE knockout abolished TGFb’s effects on cell migration, independent approaches such as knockdown of SRC expression or inhibition of SRC kinase activities in the presence of TGFb will be required to rule out potential off- target effects of CRISPR-Cas9 mediated knockout.
Response:

Thank you for this helpful suggestion. As suggested, we performed a wound-healing assay using the SRC kinase inhibitor dasatinib. Dasatinib inhibited the SRC activity in both basal state and post-TGF-β stimulation without affecting TGF-β-induced SRC expression (Fig 6C). In the presence of dasatinib, cell migration was inhibited in the presence or absence of TGF-β, suggesting that SRC activation following TGF-β-induced SRC expression was essential for cell migration (Fig S4A and B). Importantly, basal SRC activity was not significantly changed by TSE knockout (Fig 5B and D), nor was cell motility affected in the absence of TGF-β (Fig 7G and Fig S4E). These results suggest that TSE-mediated SRC expression and activation are essential for cell migration in MCF10A. We included this information in the revised manuscript. (page 10, para 2)

2.Similarly, although SRC/FAK interaction and their roles in cell migration have been well documented, it would be important to establish that FAK is indeed responsible for the observed migration phenotype in this particular system.
Response:

We appreciate the reviewer’s thoughtful comments. We agree and have performed a wound-healing assay to estimate the contribution of FAK to cell migration (Fig S4A and B). In the presence of the FAK inhibitor, Y15, cell migration was inhibited in the presence or absence of TGF-β, suggesting that FAK activity is essential for cell migration in MCF10A. (page 10, para 2)

3.According to the authors, SRC activation represents a downstream effect of TGFb that separates EMT markers (e.g., E-cadherin) from migration. This is potentially very interesting and should be further investigated. Therefore, the authors are highly encouraged to further the studies along this direction. For instance, will TSE knockout result in more “collective migration” of cell clusters in a 3D system?
Response:
We thank the reviewer for their helpful suggestions. As mentioned in Question 1, SRC activity is essential for EMT-induced cell migration. Therefore, we performed additional experiments to evaluate the effects of SRC inhibition on EMT marker expression (Fig 6C) and cell morphology (Fig 6D and E) induced by TGF-β stimulation. Notably, the SRC inhibitor did not significantly affect the repression of E-cadherin or induction of vimentin expression, and this immunoblot result is consistent with our findings in the TSE-deficient cell line (Fig 6A). Regarding the E-cadherin expression, a previous study showed that SMAD complex and SNAI1 directly bind to the E-cadherin promoter and repress its expression (Vincent et al., 2009). We also found that TGF-β stimulation induced fibroblast-like morphological change in the presence of dasatinib (Fig 6D). Furthermore, TGF-β-induced reduction of E-cadherin-mediated cell-cell adhesion was also observed even when SRC activity was inhibited (Fig 6E). Importantly, we also found E-cadherin accumulation at the contact site of the cells (Fig 6E, white arrowheads in the middle right panel). Previous studies have consistently shown that SRC- mediated endocytosis and degradation are required for the distribution of E-cadherin to the epithelial cell membrane (Balzac et al., 2005; Fujita et al., 2002). Taken together, it is likely that the expression of E-cadherin is more strongly regulated by TGF-β signalling, however, SRC activity is essential for loosening E-cadherin-mediated cell-cell adhesion. Because EMT-associated morphological changes and cell motility are closely linked, further investigation is needed to clarify the relationship between these phenomena.
As suggested by the reviewer, we performed TGF-β stimulation using a 3D culture system. However, to date, we have not found noticeable differences between the WT and TSE mutants; therefore, we would like to investigate this in our future study. We have included this discussion in the revised manuscript.(page 8, para 2)

© 2023. Published by The Company of Biologists under the terms of the Creative Commons Attribution License (https://creativecommons.org/licenses/by/4.0/).

Second decision letter

MS ID#: JOCES/2023/261001

MS TITLE: A novel TGF-&[beta]-responsive enhancer regulates SRC expression and epithelial-mesenchymal transition-associated cell migration

AUTHORS: Soshi Noshita, Yuki Kubo, Kentaro Kajiwara, Daisuke Okuzaki, Shigeyuki Nada, and Masato Okada
ARTICLE TYPE: Research Article

I am happy to tell you that your manuscript has been accepted for publication in Journal of Cell Science, pending standard ethics checks.

Reviewer 1

Advance summary and potential significance to field

In the present study, the authors examined the role of SRC induction in TGF-ß-induced EMT. They identified an enhancer responsible for the induction of SRC by TGF-ß, and inactivated the enhancer by genome editing. Using the enhancer-inactivated cells, they showed that upregulation of SRC is essential for activation of FAK and promotion of cell motility in TGF-ß-stimulated cells. This is a nice strategy to unveil the role of SRC downstream of TGF-ß. Recently, Yakymovych et al. reported the role of SRC in TGF-ß-induced FAK activation and cell motility (PMID: 36378749), with a different mode of SRC activation by TGF-ß. The authors clarified and discussed apparent discrepancy between two works. I am confident that this paper would contribute to the molecular mechanisms underlying induction of EMT.

Comments for the author

The authors sincerely addressed all of my concerns. I have no additional comments.

Reviewer 2

Advance summary and potential significance to field

Their revised manuscript has been improved according to the referee's comments.
There are no more concerns.

Comments for the author

Their revised manuscript has been improved according to the referee's comments.
There are no more concerns.

Reviewer 3

Advance summary and potential significance to field

The authors have adequately addressed my previous points.

Comments for the author

All my suggestions have been addressed.

© 2023. Published by The Company of Biologists under the terms of the Creative Commons Attribution License (https://creativecommons.org/licenses/by/4.0/).
